# Liposomal oncolytic adenovirus as a neoadjuvant therapy for triple-negative breast cancer

**DOI:** 10.1038/s41598-025-00211-2

**Published:** 2025-05-14

**Authors:** Jaimin R. Shah, Tao Dong, Abraham T. Phung, Sohini Khan, Omonigho Aisagbonhi, Sarah L. Blair, Michael Bouvet, William C. Trogler, Andrew C. Kummel

**Affiliations:** 1 Moores Cancer Center, University of California San Diego, La Jolla, CA 92093 USA; 2 Department of Chemistry & Biochemistry, University of California San Diego, La Jolla, CA 92093 USA; 3 Program in Materials Science and Engineering, University of California San Diego, La Jolla, CA 92093 USA; 4 Department of NanoEngineering, University of California San Diego, La Jolla, CA 92093 USA; 5 Department of Surgery, University of California San Diego, La Jolla, CA 92093 USA; 6 Department of Pathology, University of California San Diego, La Jolla, CA 92093 USA

**Keywords:** Triple-negative breast cancer, Neoadjuvant therapy, Oncolytic adenovirus, Coxsackievirus and adenovirus receptor, Liposomes, Metastasis, Breast cancer, Surgical oncology, Breast cancer, Drug delivery, Gene therapy

## Abstract

Breast cancer remains one of the leading causes of cancer-related death, with triple-negative breast cancer (TNBC) accounting for 15–20% of cases. TNBC, characterized by the absence of ER, PR, and HER2 protein, is an aggressive form of breast cancer that is unresponsive to hormonal therapies and HER2-targeted treatments, with fewer treatment options and poorer prognosis. Oncolytic adenoviruses (Ad) are a potential treatment option for TNBC but require coxsackievirus and adenovirus receptors (CAR) to effectively enter and transduce cancer cells. This study investigates a novel neoadjuvant therapy to improve the efficacy of an oncolytic Ad with human telomerase reverse transcriptase (Ad-hTERT) in CAR-low TNBC tumors using folate surface-modified liposomes to enhance delivery. This therapy helps deescalate treatment by reducing or eliminating the need for checkpoint inhibitors or toxic chemotherapy combinations. In vitro studies using CAR-low TNBC murine 4T1-eGFP cells, CAR-high TNBC human MDA-MB-231-GFP cells and several other TNBC human cancer cell lines with varying CAR expression demonstrated significantly higher cytotoxicity with encapsulated Ad-hTERT compared to Ad-hTERT. Similar results were observed in patient-derived primary TNBC cells. In vivo studies in immunocompetent mice with CAR-low 4T1-eGFP tumors revealed that encapsulated Ad-hTERT, administered as neoadjuvant therapy, resulted in stable or reduced tumor sizes, improved survival rates, higher apoptosis of cancer cells, lower cancer cell proliferation, and increased T-cell infiltration in resected tumors. Furthermore, encapsulated Ad-hTERT prevented lung metastasis and tumor recurrence at the primary site, resulting in higher survival rates in mice. Thus, liposomal encapsulation of Ad may be a viable strategy for treating TNBC.

## Introduction

Breast cancer remains one of the leading causes of cancer-related death in the United States. It is estimated that there will be 313,510 new cases of breast cancer and 42,780 deaths from the disease in 2024^1^. Triple-negative breast cancer (TNBC) accounts for about 15–20% of all breast cancer cases^2^. TNBC is higher among women under 40, Black women, and those with a BRCA1 mutation^3–6^. TNBC is characterized by the absence of estrogen receptors, progesterone receptors, and HER2 protein, thereby unresponsive to hormonal therapies and HER2-targeted treatments. Consequently, TNBC tends to grow and spread more rapidly, has fewer treatment options, and is associated with a poorer prognosis compared to other types of invasive breast cancer^7^. TNBC also has a higher likelihood of early recurrence and distant metastasis, particularly to the brain and lungs, with relapse rates peaking between three to five years post-surgery^8,9^.

Neoadjuvant therapy involves treatment prior to the main treatment (usually surgery) and is usually employed for TNBC. Neoadjuvant chemotherapy regimens for TNBC typically include anthracyclines (e.g., doxorubicin), taxanes (e.g., paclitaxel), and sometimes platinum-based drugs (e.g., carboplatin)^10–13^. Pembrolizumab, a checkpoint inhibitor approved by the United States food and drug administration (USFDA) in 2021, is used in combination with chemotherapy for the treatment of patients with high-risk early-stage TNBC^14,15^. It is also used before chemotherapy for patients with unresectable or metastatic TNBC that tests positive for PD-L1, as well as for advanced TNBC with high PD-L1 expression^14,16,17^. In March 2019, atezolizumab received accelerated USFDA approval for use with nab-paclitaxel in PD-L1-positive TNBC^18^. However, Roche withdrew this indication after a clinical trial (NCT03125902) failed to demonstrate progression-free survival superiority or a survival advantage, leading to the withdrawal of the approval^19,20^.

One promising approach to treating solid tumors prone to metastasis involves oncolytic viruses, which selectively replicate in cancer cells and induce immunogenic cell death. Adenoviruses (Ad), particularly subtype 5, have been developed for gene therapy and as oncolytic agents due to their genetic engineering flexibility and proven safety^21,22^. Numerous clinical trials are ongoing to evaluate the efficacy of oncolytic Ad used either alone or in combination with multi-agent cancer therapies^23^. Additionally, China’s State Food and Drug Administration has approved oncolytic Ad therapies in combination with chemotherapy for the treatment of head and neck cancer^24–26^. These viruses require coxsackievirus and adenovirus receptors (CAR) to effectively enter and transduce cancer cells. However, CAR expression is highly heterogeneous across cancer types and potentially limits the efficacy of Ad in CAR-low cancer cells^27,28^. Telomelysin, also known as OBP-301, is an oncolytic Ad developed by Oncolys BioPharma Inc., which uses the tumor-specific human telomerase reverse transcriptase (hTERT) gene promoter to regulate E1A and E1B genes^29,30^. hTERT is an enzyme that maintains telomere length, enabling unlimited cell division. It is highly expressed in many cancer cells but not healthy somatic cells^31,32^. Its overexpression in cancer allows these cells to bypass normal cellular aging processes, contributing to uncontrolled proliferation^32^. This oncolytic Ad with h-TERT (Ad-hTERT) demonstrated promising results in a Phase I dose-escalation study involving endoscopic intratumoral injection with radiotherapy in esophageal cancer patients (NCT03213054)^33,34^. A Phase II trial in combination with pembrolizumab for esophagogastric adenocarcinoma (NCT03921021) is ongoing.

**Fig. 1:**
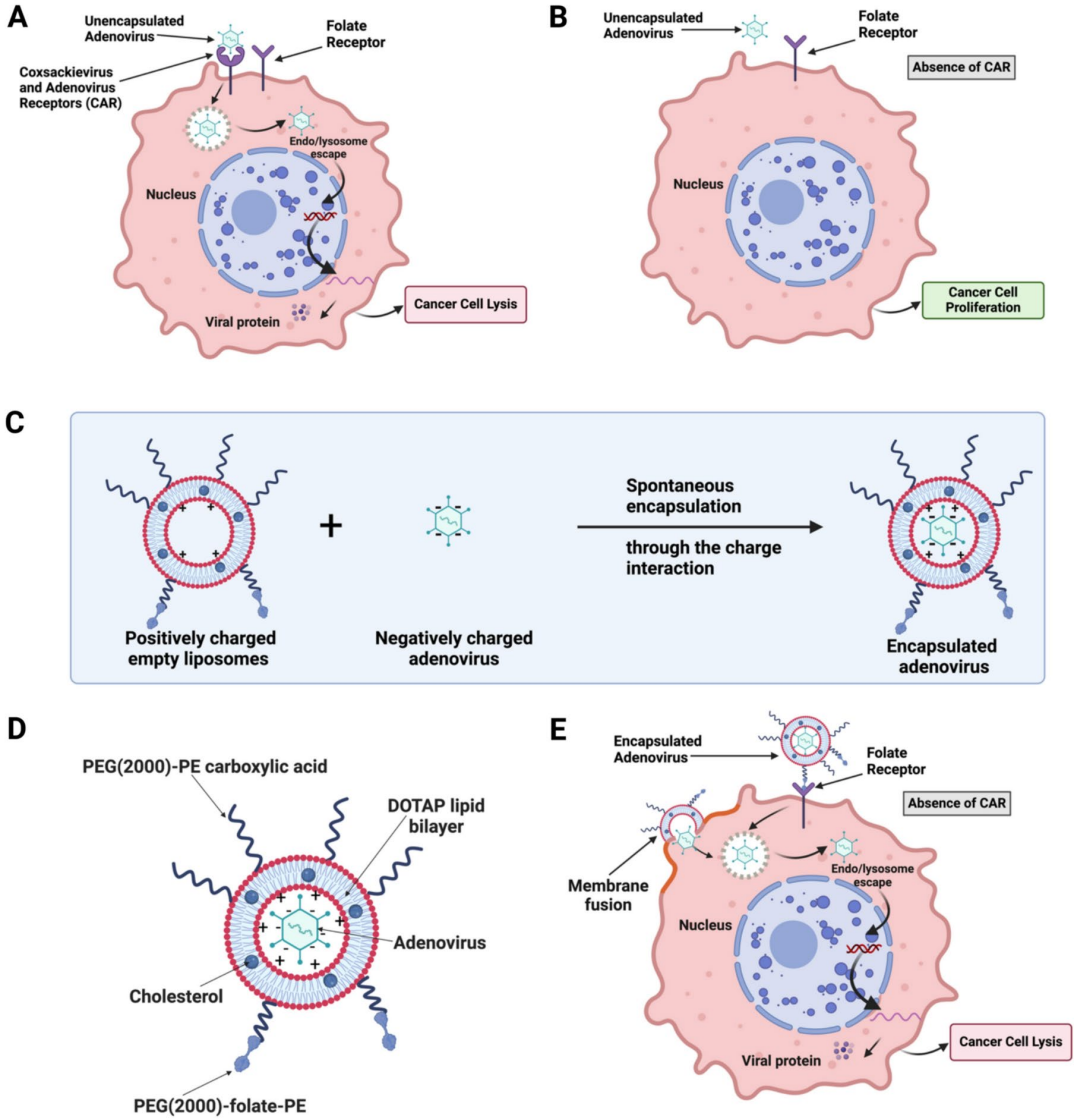
Mechanism of Ad and liposome-encapsulated Ad internalization in cancer cells. (**A**) Ad relies heavily on the coxsackievirus and adenovirus receptor (CAR) for cell entry. (**B**) Ad transduce inefficiently in cancer cells with low or absent CAR expression. Low CAR expression is notable in tumors such as TNBC, which causes resistance to Ad-based gene therapy. (**C**) The process of encapsulating Ad in liposomes: The virus becomes encapsulated within liposomes due to favorable charge interactions. (**D**) Cationic liposome containing negatively charged Ad. (**E**) Encapsulated Ad can enter tumor cells via folate receptor-mediated endocytosis and membrane fusion, even in the absence of CAR expression.

The present study explores a novel neoadjuvant therapy aimed at improving Ad efficacy in CAR-low TNBC tumors in immunocompetent BALB/c mice to prevent metastasis and recurrence. In the present study, liposomes were surface-modified with folate to enhance Ad delivery and efficacy in CAR-low cancer cells, obviating the need for checkpoint inhibitors or toxic chemotherapy combinations. Folate receptors are highly selective tumor markers overexpressed in many human cancers, including brain, kidney, breast, and lung^35^. Negatively charged Ad is encapsulated within positively charged liposomes through charge interactions^36^. Liposomal encapsulated Ad can enter cells via both folate receptor-mediated endocytosis and membrane fusion, even in the absence of CAR expression^36,37^ (Fig. 1). In the present study, Ad-hTERT was manufactured and encapsulated in liposomes to enhance its efficacy against TNBC. In vitro studies on CAR-low TNBC murine mammary carcinoma 4T1 cells with enhanced green fluorescent protein (4T1-eGFP), CAR-high TNBC human mammary adenocarcinoma MDA-MB-231 cells with green fluorescent protein (MDA-MB-231-GFP), and several other TNBC human cancer cell lines with varying CAR expression demonstrated significantly higher cytotoxicity with encapsulated Ad-hTERT compared to Ad-hTERT. Similar results were observed in patient-derived primary TNBC cells. In vivo studies in immunocompetent mice bearing CAR-low 4T1-eGFP tumors in the mammary fat pad revealed that encapsulated Ad-hTERT, administered as a neoadjuvant therapy, resulted in stable or reduced tumor sizes, higher cancer cell apoptosis, lower cancer cell proliferation, increased T-cell infiltration in resected tumors, and improved survival rates. Encapsulated Ad-hTERT also prevented lung metastasis and tumor recurrence at the primary site, which shows enhanced efficacy and the potential for treating aggressive TNBC with CAR heterogeneity.

## Results and discussion

### Comparative in vitro transduction of Ad-hTERT and encapsulated Ad-hTERT in TNBC cells

**Fig. 2:**
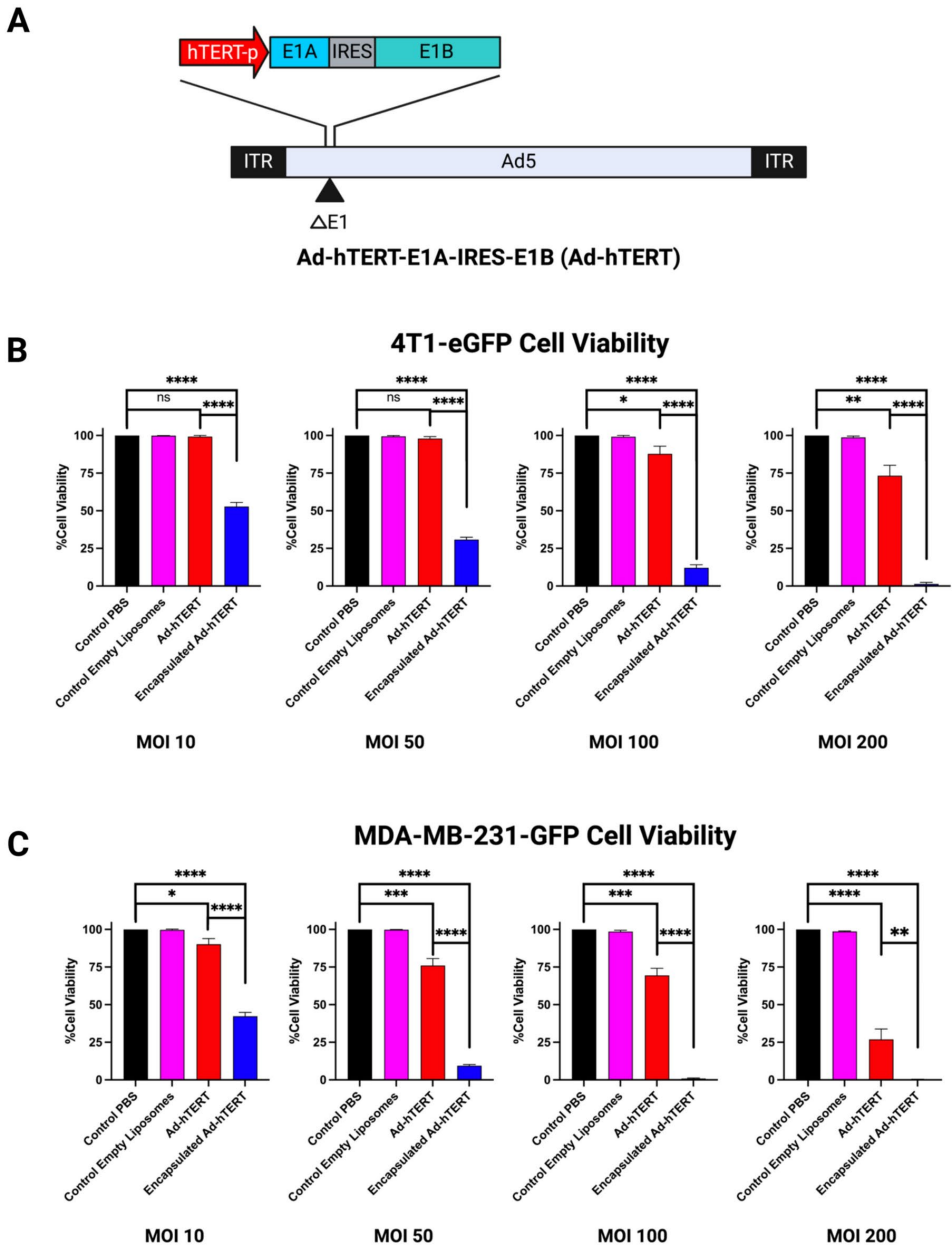
In vitro transduction of Ad-hTERT and encapsulated Ad-hTERT in TNBC cells. (**A**) Schematic of Ad-hTERT, a telomerase-specific, replication-competent adenovirus with E1A and E1B genes driven by the hTERT promoter and linked via an IRES. (**B**) Cell viability of 4T1-eGFP cells treated with Ad-hTERT (red) and encapsulated (blue) Ad-hTERT at various MOIs for 72 hours (n = 3). Encapsulated Ad-hTERT showed significantly higher cytotoxicity at MOI 10, 50, 100, and 200 (****p ≤ 0.0001). (**C**) Cell viability of MDA-MB-231-GFP cells under identical conditions (n = 3). Encapsulated Ad-hTERT demonstrated significantly higher cytotoxicity at MOIs of 10, 50, and 100 (****p ≤ 0.0001), and at an MOI of 200 (**p ≤ 0.01). In (**C**), the blue bar is not visible due to near-complete cell death.

**Fig. 3:**
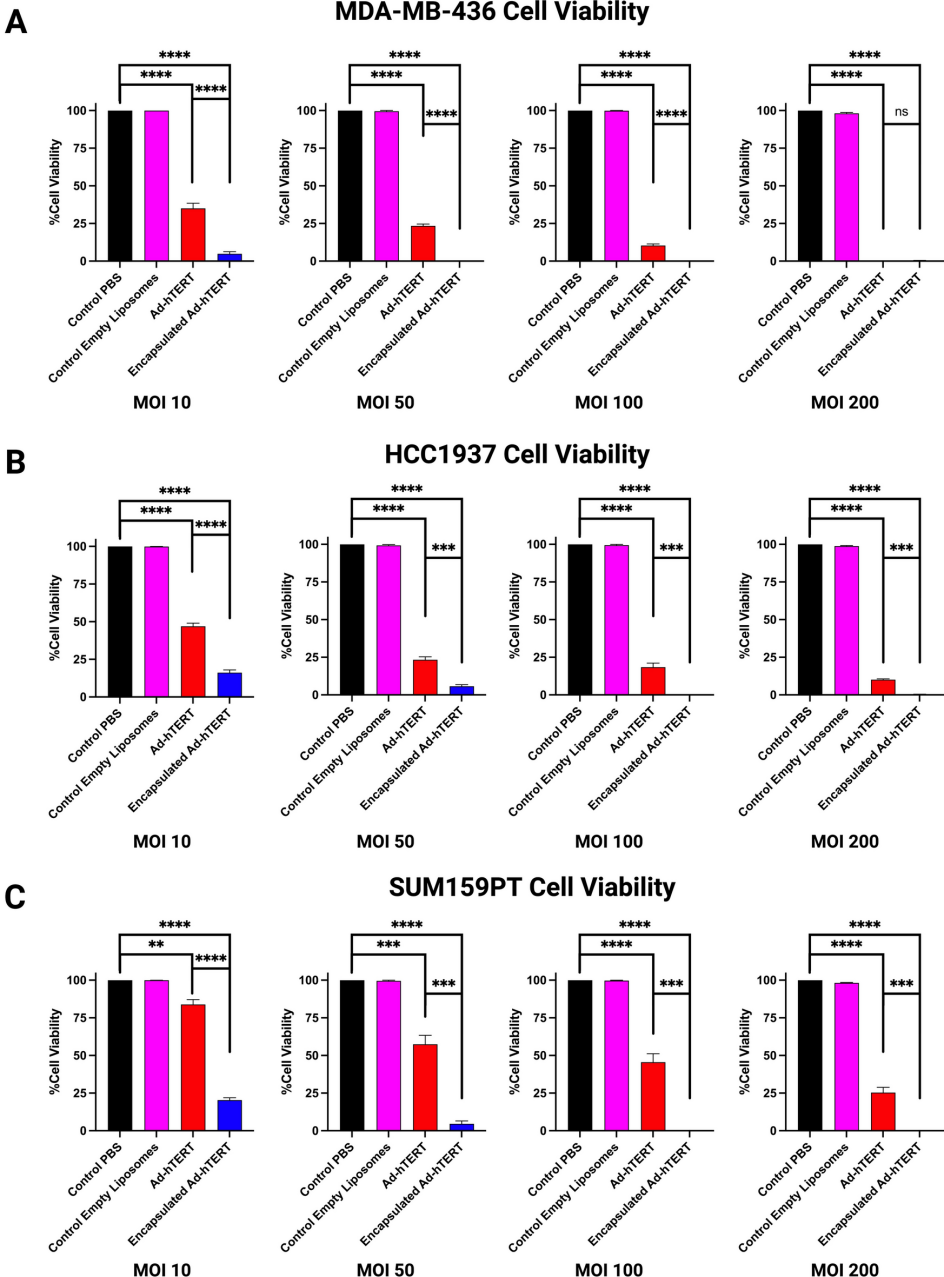
In vitro transduction of Ad-hTERT and encapsulated Ad-hTERT in additional TNBC cell lines. (**A**–**C**) Cell viability of MDA-MB-436, HCC1937, and SUM159PT cells, respectively, following treatment with Ad-hTERT (red) and encapsulated (blue) Ad-hTERT at various MOIs for 72 hours (n = 3). Encapsulated Ad-hTERT exhibited significantly higher cytotoxicity at MOI 10 and 100 in all cell lines: MDA-MB-436 (****p ≤ 0.0001), HCC1937 (***p ≤ 0.001), and SUM159PT (***p ≤ 0.001). .

To confirm the specificity of Ad-hTERT (Fig. 2A) it was compared with wild type Ad (Ad-WT) in both TERT-positive A549 cells (human adenocarcinoma alveolar basal epithelial cells), and TERT-negative CCD 18Lu (lung fibroblast) cells. Cytotoxicity of Ad-hTERT was specific to the TERT-positive A549 cells (Fig. S1). Immunofluorescent CAR staining was performed on 4T1-eGFP (murine mammary carcinoma), MDA-MB-231-GFP (human breast adenocarcinoma), MDA-MB-436 (human breast adenocarcinoma), HCC1937 (human breast carcinoma) and SUM159PT (anaplastic carcinoma of the breast) TNBC cells (Figs. S2, S3). The 4T1-eGFP and SUM159PT cells were identified as CAR-low, exhibiting weak CAR positivity, while the MDA-MB-231-GFP, MDA-MB-436, and HCC1937 cells were identified as CAR-high, showing stronger CAR positivity. All cell lines were transduced at a multiplicity of infection (MOI) of 10, 50, 100, and 200 with control PBS, control empty liposomes (at the dose equivalent to encapsulated Ad-hTERT at respective MOI), Ad-hTERT, and encapsulated Ad-hTERT at varying MOIs and cell viability was performed after 72 hours (n = 3) (Figs. 2, 3). Comparative quantitative percentage cell viability illustrates that encapsulated Ad-hTERT exhibited significantly higher cytotoxicity compared to Ad-hTERT in all TNBC cells varying in CAR expressions. Encapsulated Ad-hTERT exhibited higher cytotoxic efficacy in CAR-low murine, CAR-low human, and CAR-high human TNBC cells when treated for 72 hours at various low to high MOIs. The empty liposome control did not show a cytotoxic effect compared to the PBS control. The physicochemical characterization of liposomes is presented in (Table S1). Comparative GFP fluorescence microscopy images of 4T1-eGFP and MDA-MB-231-GFP cells were reported (Fig. S4). As shown in (Fig. S5), experimental replicates confirmed the reproducibility of the findings. Additionally, (Fig. S6) provides evidence supporting folate receptor-mediated endocytosis.

### Comparative in vitro transduction of Ad-hTERT and encapsulated Ad-hTERT in patient-derived primary TNBC cells

**Fig. 4:**
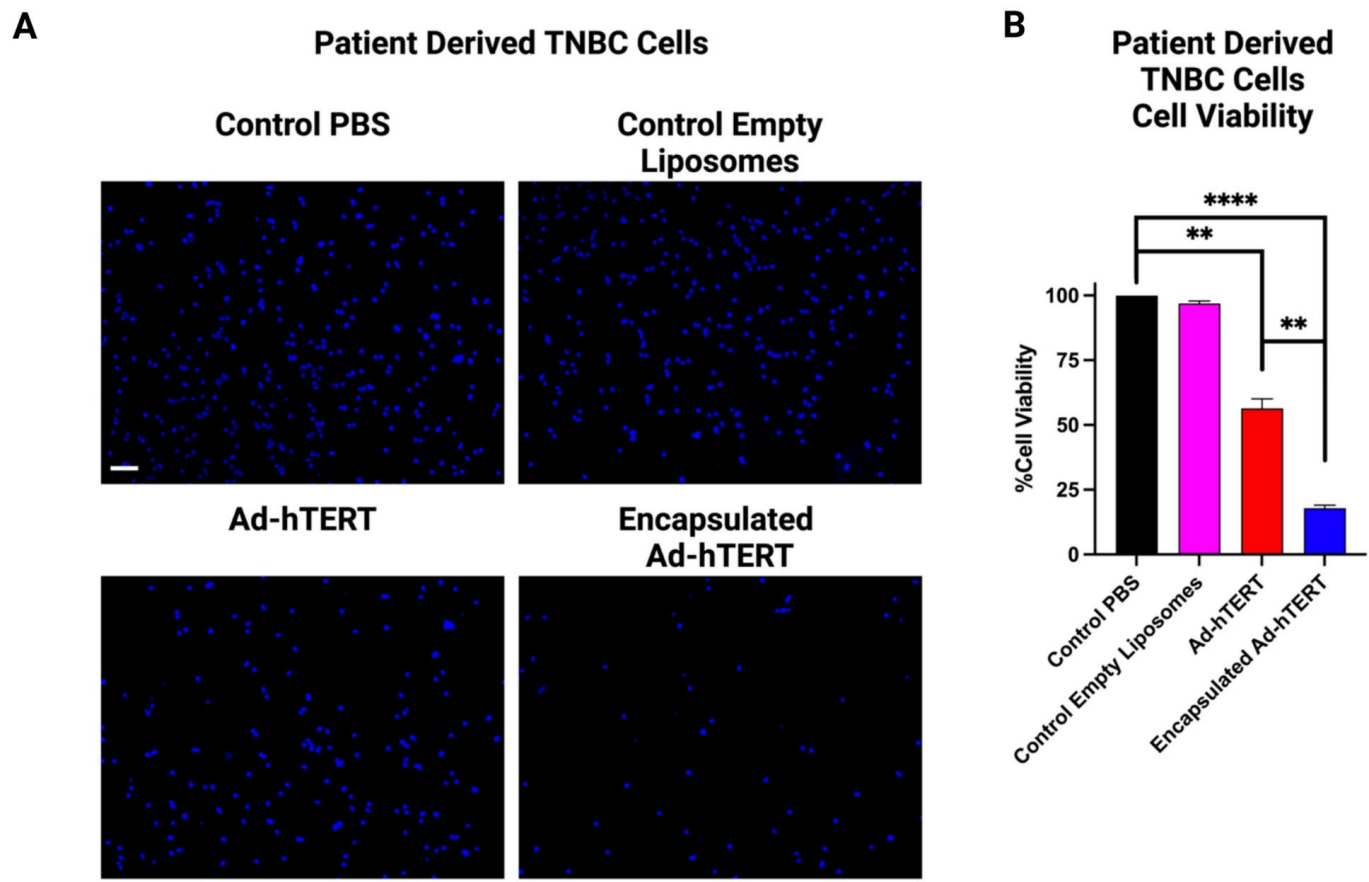
In vitro transduction of Ad-hTERT and encapsulated Ad-hTERT in patient-derived primary TNBC cells. (**A**) DAPI-stained fluorescence microscopy images of cells treated with Ad-hTERT and encapsulated Ad-hTERT at MOI 100 for 72 hours. Scale bar = 100 μm. (**B**) Cell viability of patient-derived primary TNBC cells treated with Ad-hTERT (red) and encapsulated (blue) Ad-hTERT under the same conditions (n = 3). Encapsulated Ad-hTERT induced significantly higher cytotoxicity (**p ≤ 0.01).

To investigate the transduction efficiency of Ad-hTERT and encapsulated Ad-hTERT in patient-derived primary TNBC cells, a TNBC tumor was digested and homogenized into a single-cell suspension. These cells were transduced with control PBS, control empty liposomes, Ad-hTERT, and encapsulated Ad-hTERT at an MOI of 100 for 72 hours (n = 3). Comparative DAPI-stained fluorescence microscopy images (Fig. 4A) and quantitative percentage cell viability data (Fig. 4B) revealed that both Ad-hTERT and encapsulated Ad-hTERT exhibited higher cytotoxicity compared to PBS-treated control cells. Specifically, Ad-hTERT showed a notable increase in cytotoxicity (p-value: ** ≤ 0.01), while encapsulated Ad-hTERT exhibited even greater cytotoxic effects (p-value: **** ≤ 0.0001). Furthermore, encapsulated Ad-hTERT demonstrated higher cytotoxicity compared to Ad-hTERT (p-value: ** ≤ 0.01). These results suggest that encapsulated Ad-hTERT can be significantly more effective in these patient-derived primary TNBC cells compared to Ad-hTERT.

### Comparative in vivo biodistribution of Ad-hTERT and encapsulated Ad-hTERT in immunocompetent mice bearing CAR-low 4T1-eGFP TNBC tumors

**Fig. 5:**
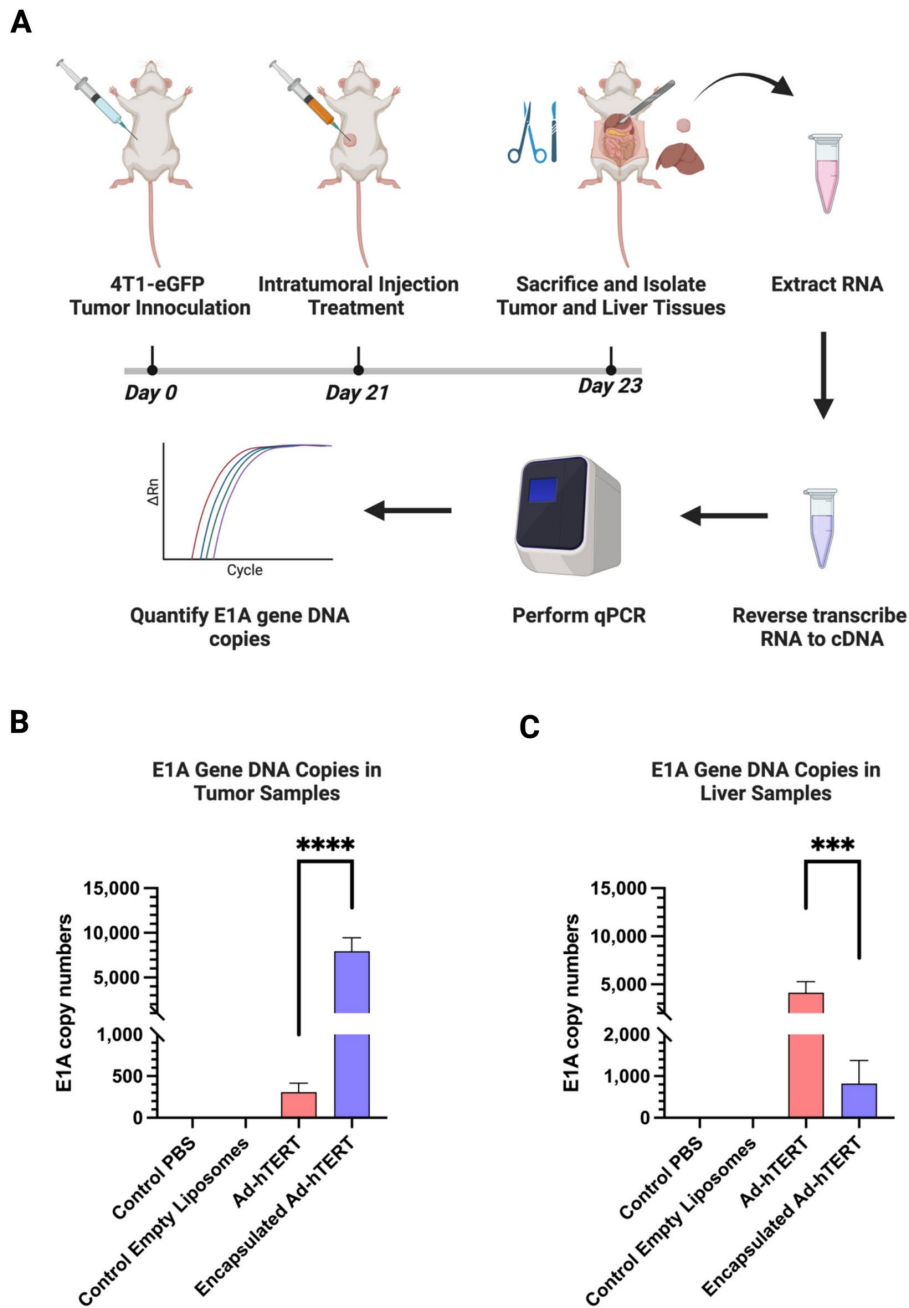
Comparative in vivo biodistribution of Ad-hTERT and encapsulated Ad-hTERT on CAR-low 4T1-eGFP TNBC tumors. (**A**) Treatment model: CAR-low 1 × 10^6^ 4T1-eGFP TNBC tumors were inoculated into the mammary fat pad of BALB/c mice. The tumors were treated with intratumoral injection of PBS, empty liposomes, Ad-hTERT, or encapsulated Ad-hTERT as neoadjuvant therapy. 2 days after the treatment, mice were sacrificed and liver and tumor tissues were isolated for E1A gene copy numbers vis RT-qPCR (**B**) E1A gene copy numbers in tumors treated with PBS (n = 3), control empty liposomes (n = 3), Ad-hTERT (n = 5), and encapsulated Ad-hTERT (n = 5). Encapsulated Ad-hTERT demonstrated significantly higher E1A copy numbers (p-value: **** ≤ 0.0001) compared to the Ad-hTERT. (**C**) E1A gene copy numbers in liver tissues demonstrated significantly lower E1A copy numbers (p-value: *** ≤ 0.001) in mice treated with encapsulated AD-hTERT compared to the Ad-hTERT.

The biodistribution of Ad-hTERT and encapsulated Ad-hTERT were investigated in immunocompetent BALB/c mice bearing CAR-low 4T1-eGFP TNBC tumors in the mammary fat pad (Fig. 5A). Mice were subcutaneously inoculated with 1 × 10^6^ 4T1-eGFP cells in the fat pad. Tumor volume was monitored until it reached approximately 30 mm^3^, at which point treatment was performed. Four treatment groups were used to evaluate the therapeutic effect of encapsulation on CAR-low tumors: control PBS (n = 3), control empty liposomes (at the dose equivalent to encapsulated Ad-hTERT) (n = 3), Ad-hTERT (n = 5), and encapsulated Ad-hTERT (n = 5). RT-qPCR was performed to quantify the E1A gene copy numbers. With CAR-low 4T1-eGFP tumors, Ad-hTERT intratumoral injection resulted in poor transduction of the tumor (p-value: **** ≤ 0.0001) compared to the encapsulated Ad-hTERT (Fig. 5B). And primarily led to off-target transduction in the liver. Contrary to the encapsulated Ad-hTERT-treated group, off-target transduction of the liver was found to be much lower (p-value: *** ≤ 0.001) compared to the Ad-hTERT (Fig. 5C). These results demonstrated that liposomal encapsulation enhances the transduction efficiency in vivo when treated locally in CAR-low 4T1-eGFP TNBC tumors.

### Comparative in vivo therapeutic efficacy of Ad-hTERT and encapsulated Ad-hTERT in immunocompetent mice bearing CAR-low 4T1-eGFP TNBC tumors

**Fig. 6:**
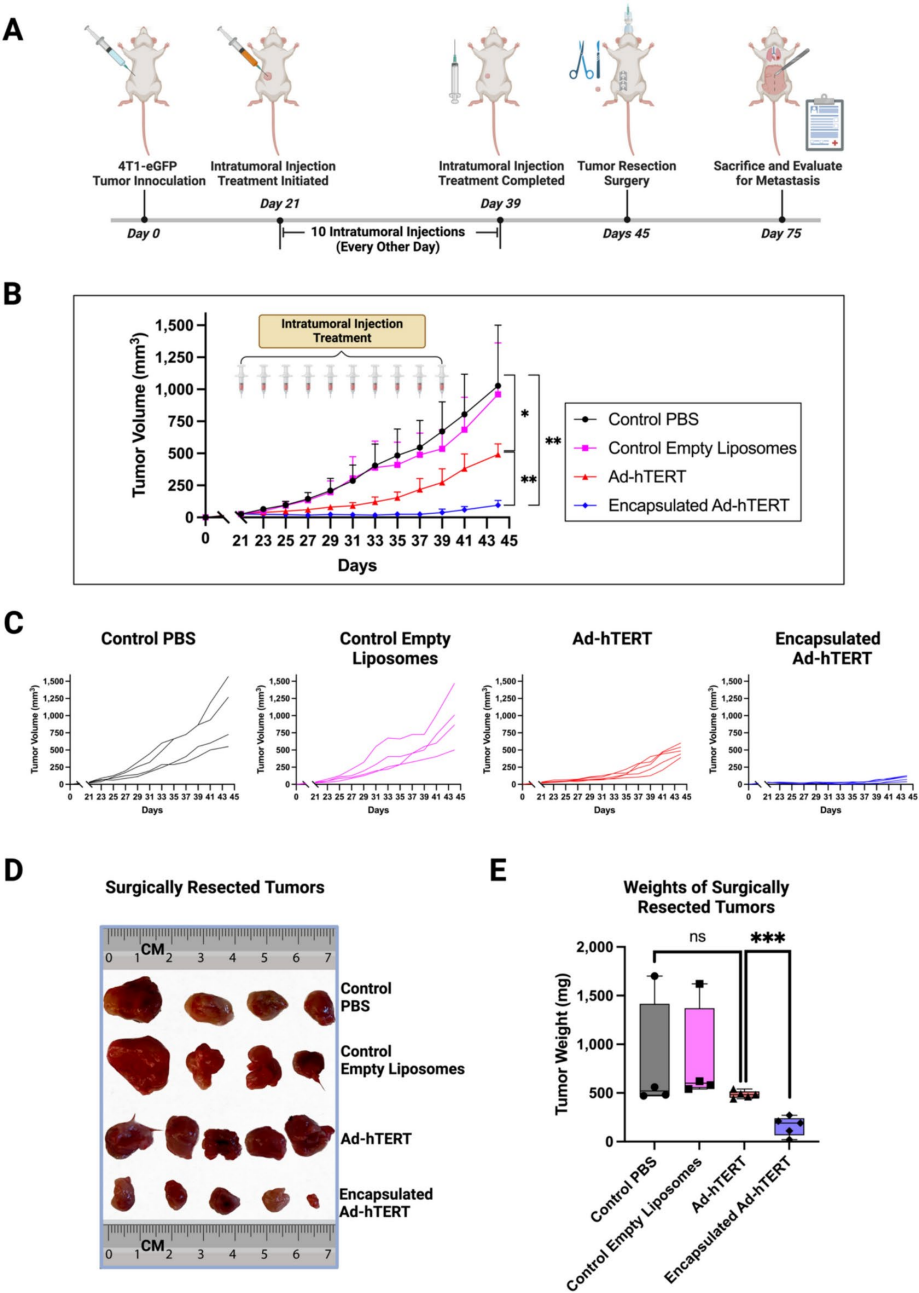
Comparative in vivo therapeutic efficacy of Ad-hTERT and encapsulated Ad-hTERT on CAR-low 4T1-eGFP TNBC tumors. (**A**) Treatment model: CAR-low 1 × 10^6^ 4T1-eGFP TNBC tumors were inoculated into the mammary fat pad of BALB/c mice. The tumors were treated with intratumoral injections of PBS, empty liposomes, Ad-hTERT, or encapsulated Ad-hTERT as neoadjuvant therapy. Upon completion of treatment, the tumors were resected, and the mice were monitored for survival probability and metastasis evaluation. (**B**) Average tumor growth curves: Tumor growth curves for mice treated with control PBS (n = 4), control empty liposomes (n = 4), Ad-hTERT (n = 5), and encapsulated Ad-hTERT (n = 5). Encapsulated Ad-hTERT demonstrated significantly higher therapeutic efficacy (p-value: ** ≤ 0.01) compared to the Ad-hTERT. (**C**) Individual tumor growth curves of each group. (**D**) Images of surgically removed tumors. (**E**) Tumor weights: Weights of surgically removed tumors upon completion of intratumoral injection treatment, shown for each treatment group. Encapsulated Ad-hTERT demonstrated significantly lower tumor weights (p-value: *** ≤ 0.001) compared to the Ad-hTERT.

The neoadjuvant therapeutic effects of Ad-hTERT and encapsulated Ad-hTERT were investigated in immunocompetent BALB/c mice bearing CAR-low 4T1-eGFP TNBC tumors in the mammary fat pad. Mice were subcutaneously inoculated with 1 × 10^6^ 4T1-eGFP cells in the fat pad. Tumor volume was monitored until it reached approximately 30 mm^3^, at which point treatment was initiated. Four treatment groups were used to evaluate the therapeutic effect of encapsulation on CAR-low tumors: control PBS (n = 4), control empty liposomes (at the dose equivalent to encapsulated Ad-hTERT) (n = 4), Ad-hTERT (n = 5), and encapsulated Ad-hTERT (n = 5). While human Ads do not reproduce well in murine cells, they can selectively cause dose-dependent cytotoxicity in murine cancer cells, making murine models valuable for testing^38^. The agents were intratumorally injected every other day for ten doses (Fig. 6A). Each dose was 100 µL, containing either 1.0 × 10^8^ plaque-forming units (PFU) of Ad-hTERT or encapsulated Ad-hTERT. Tumor volume was monitored every other day throughout the treatment period (Fig. 6B,C).

After completion of the neoadjuvant intratumoral therapy, Ad-hTERT-treated tumors showed regression compared to control PBS-treated tumors (p-value: * ≤ 0.05). Encapsulated Ad-hTERT-treated tumors showed even greater regression compared to control PBS-treated tumors (p-value: ** ≤ 0.01). Furthermore, encapsulated Ad-hTERT demonstrated significantly more effective tumor regression compared to Ad-hTERT (p-value: ** ≤ 0.01).

On Day 45, tumors were resected (Fig. 6D), weighed, and mice were monitored for survival probability over the next 30 days post-resection. Encapsulated Ad-hTERT-treated tumors demonstrated significantly lower weights (p-value: *** ≤ 0.001) compared to Ad-hTERT-treated tumors (Fig. 6E). This data indicates a substantial improvement in therapeutic outcomes with encapsulated Ad-hTERT.

**Fig. 7:**
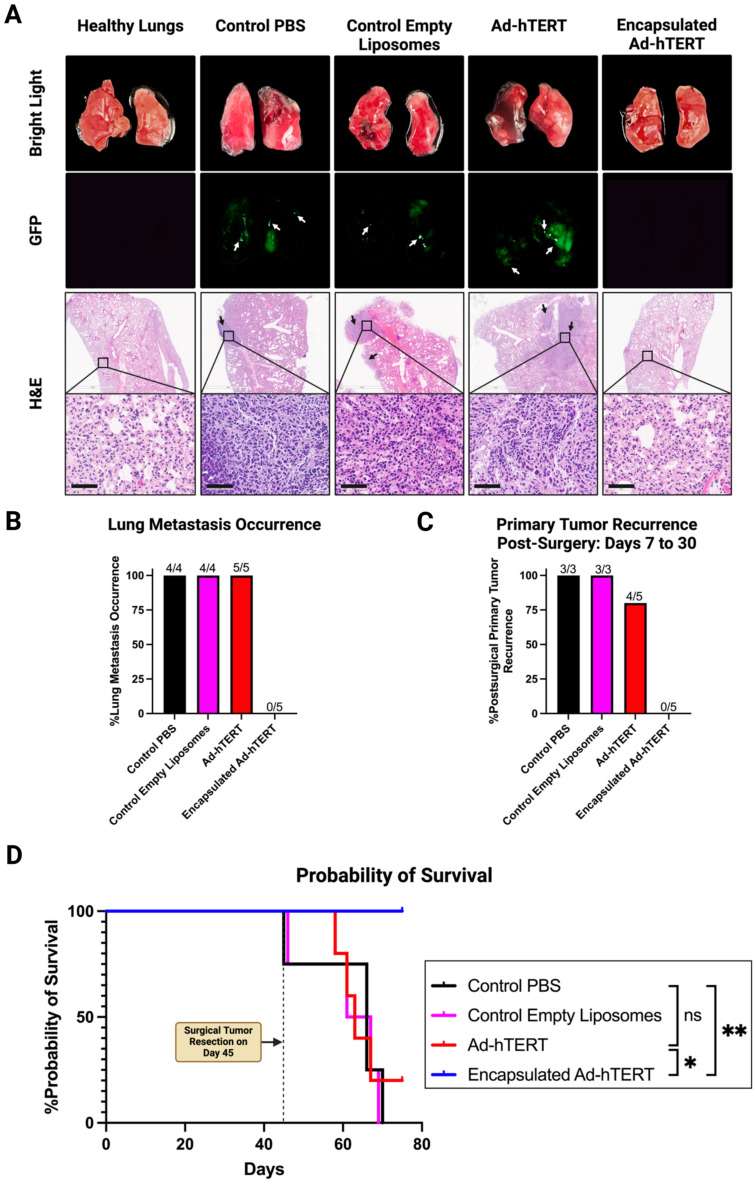
Comparative in vivo therapeutic efficacy of Ad-hTERT and encapsulated Ad-hTERT on CAR-low 4T1-eGFP TNBC tumors recurrence and lung metastases in immunocompetent mice. (**A**) Representative bright-field images, Dino-Lite GFP digital microscope images, and H&E-stained images of isolated lungs. White arrows in the GFP images and black arrows in the H&E-stained images indicate metastases present only in the lungs of the control and Ad-hTERT groups. Scale bar = 60 μm. (**B**) Lung metastasis was observed in all groups except the mice treated with encapsulated Ad-hTERT. (**C**) After 1 week of tumor resection and up to 30 days post-surgery, primary murine tumor recurrence was observed in all groups except mice treated with encapsulated Ad-hTERT. (**D**) Survival curves for mice treated with control PBS (n = 4), control empty liposomes (n = 4), Ad-hTERT (n = 5), and encapsulated Ad-hTERT (n = 5). Encapsulated Ad-hTERT demonstrated significantly higher survival probability compared to Ad-hTERT (p-value: * ≤ 0.05) and control PBS (p-value: ** ≤ 0.01).

On Day 75, surviving mice were sacrificed, and their lungs, along with the preserved lungs from mice that did not survive, were isolated to study metastasis. 4T1 TNBC tumors inoculated in the mammary fat pad are known to metastasize to the lungs^39,40^. Bright light images, Dino-Lite GFP digital microscope images, and Hematoxylin and eosin (H&E) staining images of isolated lungs from the control and treatment groups were compared to those from healthy lungs (Fig. 7A). Lungs from healthy mice and all (5/5; 100%) mice treated with encapsulated Ad-hTERT showed no GFP signal and no metastases in H&E-stained images, indicating the absence of metastatic spread. Conversely, lungs from 4/4 (100%) PBS-treated control mice, 4/4 (100%) empty liposome-treated control mice, and 5/5 (100%) Ad-hTERT-treated mice exhibited 4T1-eGFP metastases in both GFP and H&E-stained images (Fig. 7B). Post-surgery, tumor recurrence at the primary site was observed between 7 and 30 days. One mouse in each control group did not survive the surgery and died during recovery. Therefore, only three surviving mice in each control group were examined for recurrence. Recurrence was observed in 3/3 (100%) of the control PBS-treated mice, 3/3 (100%) of the control empty liposomes-treated mice, 4/5 (80%) of the Ad-hTERT-treated mice, and 0/5 (0%) of the encapsulated Ad-hTERT-treated mice (Fig. 7C).

During the post-surgery period, 100% of the control PBS-treated mice and 100% of the control empty liposome-treated mice did not survive. In contrast, 20% of the Ad-hTERT-treated mice, and 100% of the encapsulated Ad-hTERT-treated mice survived (p-value: ** ≤ 0.01 compared to control PBS, * ≤ 0.05 compared to Ad-hTERT) (Fig. 7D).

These results suggest that encapsulated Ad-hTERT is significantly more effective in preventing tumor recurrence and metastasis compared to Ad-hTERT, and it significantly improves survival probability.

### Caspase-3, Ki-67, CD3, and CD8 staining of primary murine tumors

**Fig. 8:**
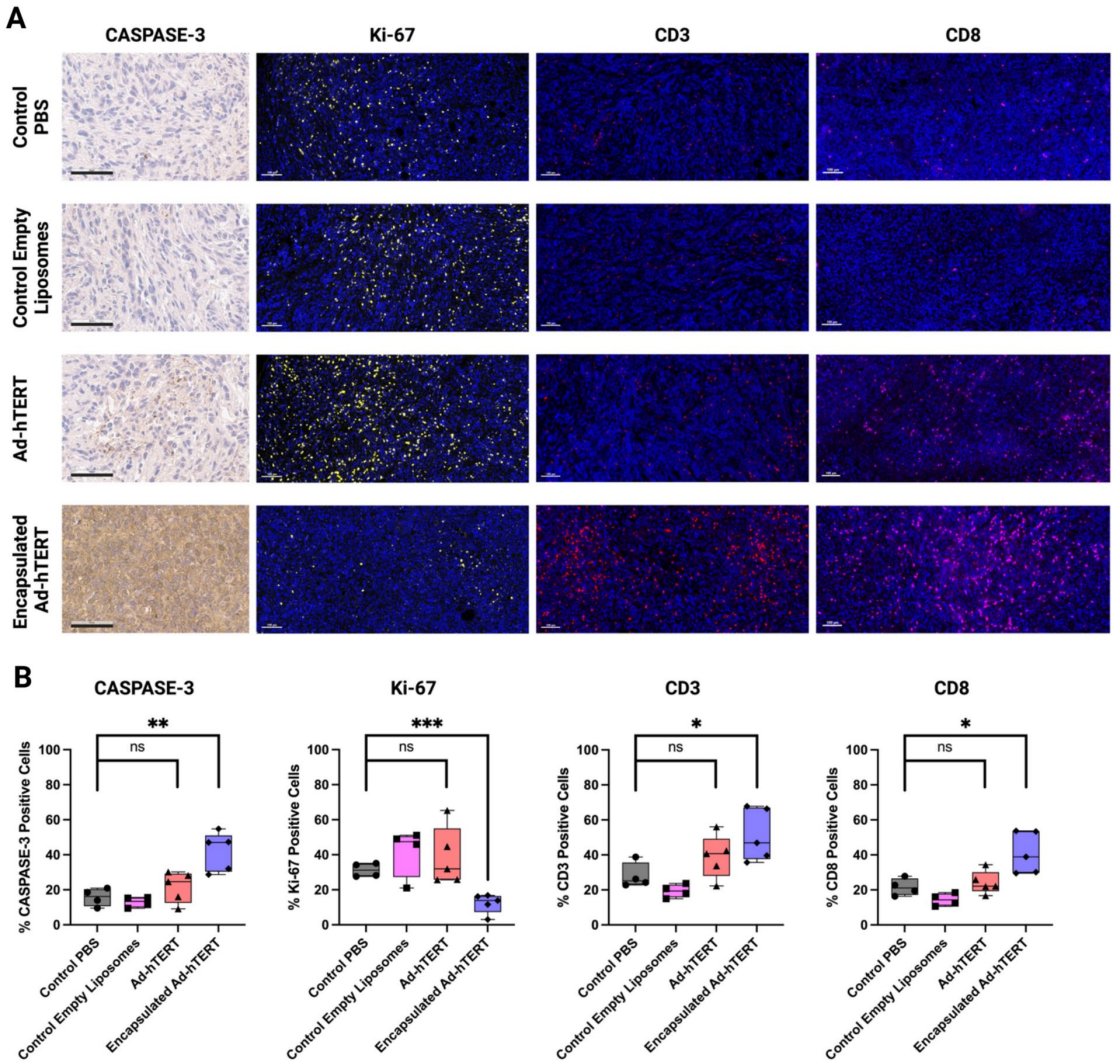
Caspase-3 IHC, Ki-67 IF, CD3 IF, and CD8 IF staining of primary murine tumors: (**A**) Caspase-3 IHC, Ki-67 IF, CD3 IF, and CD8 IF staining of primary murine tumors. IF staining: Immunofluorescent microscopy images show DAPI-stained cells are blue, Ki-67-stained cells are yellow, CD3-stained cells are red, and CD8-stained cells are magenta. Black scale bar = 60 μm, and white scale bar = 100 μm. (**B**) Comparative quantitative analysis of caspase-3, Ki-67, CD3, and CD8 positive cells in control PBS (n = 4), control empty liposomes (n = 4), Ad-hTERT (n = 5), and encapsulated Ad-hTERT (n = 5). The entire tumor sections were used for calculation. The percentage of caspase-3-positive cells was calculated relative to hematoxylin-positive cells. The Ki-67, CD3, and CD8 positive cell percentages were calculated relative to DAPI-positive cells.

To elucidate the impact of encapsulation on tumor proliferation, apoptosis, and immune cell infiltration, immunohistochemical staining for caspase-3 (an apoptosis marker) was performed, and immunofluorescent staining for Ki-67 (a proliferation marker), CD3 (a T-cell marker), and CD8 (a cytotoxic T-cell marker) was performed. H&E staining of primary murine tumors was also performed, as shown in (Fig. S7). Caspase-3 staining revealed a low number of positive cells in tumors treated with PBS and empty liposomes, a moderate number in tumors treated with Ad-hTERT, and a significantly higher number in tumors treated with encapsulated Ad-hTERT. In contrast, immunofluorescent images showed a high number of Ki-67 positive cells in tumors treated with PBS, empty liposomes, and Ad-hTERT, whereas tumors treated with encapsulated Ad-hTERT exhibited a very low number of Ki-67 positive cells. CD3 and CD8 staining revealed a low number of positive cells in tumors treated with PBS, empty liposomes, and Ad-hTERT but a high number in tumors treated with encapsulated Ad-hTERT (Fig. 8A).

A comparative quantitative analysis of caspase-3, Ki-67, CD3, and CD8 positive cells was performed on mice treated with control PBS (n = 4), control empty liposomes (n = 4), Ad-hTERT (n = 5), and encapsulated Ad-hTERT (n = 5). The Ad-hTERT treatment group did not show a significantly higher number of caspase-3 positive cells compared to the control PBS group. However, the encapsulated Ad-hTERT treatment group demonstrated a significantly higher number of caspase-3 positive cells (**p ≤ 0.01). Similarly, while the Ad-hTERT-treated group did not show a significantly lower number of Ki-67 positive cells compared to the control PBS group, the encapsulated Ad-hTERT treatment group exhibited a significantly lower number of Ki-67 positive cells (***p ≤ 0.001). The Ad-hTERT-treated group did not show significantly higher CD3 and CD8 positive cells compared to the control PBS group. However, the encapsulated Ad-hTERT-treated group demonstrated a significantly higher number of CD3 and CD8 positive cells (*p ≤ 0.05). (Fig. 8B).

These data indicate that encapsulation enhances the apoptotic effect of Ad-hTERT while concurrently reducing tumor cell proliferation, highlighting the potential therapeutic advantage of encapsulated Ad-hTERT in tumor treatment. Additionally, the data suggest that encapsulation facilitates the infiltration of T-cells within the tumor microenvironment, potentially enhancing the immune response against the tumor.

In previous preclinical studies involving oncolytic Ad, one study compared various viral vectors, including Ad, used as neoadjuvant therapy in a murine model of TNBC. This study reported approximately 20–40% lung metastasis rates across all treatment groups^41^. Another study demonstrated the antitumor efficacy of Ad treatment; however, it did not report any evidence of metastasis prevention or recurrence suppression^42^. Oncolytic Ad and other types of oncolytic viral therapies for TNBC have used combination therapies to enhance antitumor activity and prevent lung metastasis. These studies have demonstrated that combining Ad with PD-L1 and CTLA-4 inhibitors results in significant antitumor and antimetastatic effects in mice TNBC tumors^43–45^. Similarly, other research has shown that combining Ad with cyclophosphamide or trichostatin leads to improved antitumor efficacy and survival rates in treated mice^46–48^. Checkpoint inhibitors or chemotherapies can cause off-target toxicities and long-term adverse effects^49–52^. Successful prevention of local and distant metastasis by type-5 Ad treatment has been demonstrated in only a relatively CAR-high tumor model in immunocompromised mice^53^. To the best of the authors’ knowledge, the present study is the first to demonstrate both antitumor and antimetastatic effects of an encapsulated Ad therapy in a CAR-low tumor model in immunocompetent mice, achieved without the use of combination chemotherapy or checkpoint inhibitors. In contrast, another study that demonstrated antitumor and antimetastatic effects in immunocompetent mice utilized vesicular stomatitis virus matrix protein-containing liposomes^54^ (Table 1).

**Table 1:** Comparison of TNBC tumor models and treatment outcomes from this study and previously published work.

No	Therapeutic agent(s)	Tumor and mouse model used	Study outcome	Reference(s)
1	Liposomal encapsulated type-5 oncolytic Ad	4T1-eGFP in mammary fat pad of immunocompetent BALB/c mice	Enhanced antitumor effect due to the neoadjuvant therapy with liposomal encapsulated Ad. Enhanced immune cell infiltration, reduced lung metastasis, reduced recurrence, and improved survival	Current study
2	Oncolytic Ad in combination with PD-L1 and anti-CTLA-4	4T1 in mammary fat pads of BALB/C mice	Antitumor and antimetastatic effect due to the combination therapy. Enhanced immune cell activation	^43^
3	Type-5 oncolytic Ad	4T1 in right flank of BALB/C mice	Antitumor effect due to the therapy	^42^
4	Type-5 oncolytic Ad and other oncolytic viruses such as herpes simplex virus	4T1 in left flank and rechallenge in second right mammary fat pad of BALB/C mice	Antitumor effect against primary murine tumor and protection against rechallenge when treated as neoadjuvant therapy. All treatment groups showed ~ 20 to 40% lung metastasis	^41^
5	Type-5 oncolytic Ad in combination with anti-PD-1	4T1 in the right flank of BALB/C mice	Antitumor effect due to the therapy	^44^
6	Type-5 oncolytic Ad	MDA-MB-231-RFP in flank of athymic nude mice	Prevention of local and distant metastasis due to therapy	^53^
7	Recombinant Ad5/Hexon Ad48 oncolytic Ad in combination with PD-L1 and anti-CTLA-4	4T1 in right flank of BALB/C mice	Antitumor and antimetastatic effect due to the therapy	^45^
8	Vesicular stomatitis virus matrix protein-containing liposomes	4T1 in right flank of BALB/C mice	Antitumor and antimetastatic effect due to the therapy	^54^
9	Type 5/type 3 oncolytic Ad in combination with Cyclophosphamide	MDA-MB-436 in the two uppermost mammary fat pads of nude mice	Antitumor efficacy was observed only in groups treated with a combination of oncolytic Ad and Cyclophosphamide	^46^
10	Type 5 oncolytic Ad in combination with Trichostatin A	SUM159 in one flank of nude mice	Improved survival was observed in groups treated with a combination of oncolytic Ad and Trichostatin A	^47^
11	Oncolytic Ad in combination with Cyclophosphamide	MDA-MB-231-LUC cells in left flank of nude mice	Antitumor efficacy was observed only in groups treated with a combination of oncolytic Ad and Cyclophosphamide	^48^

**Fig. 9:**
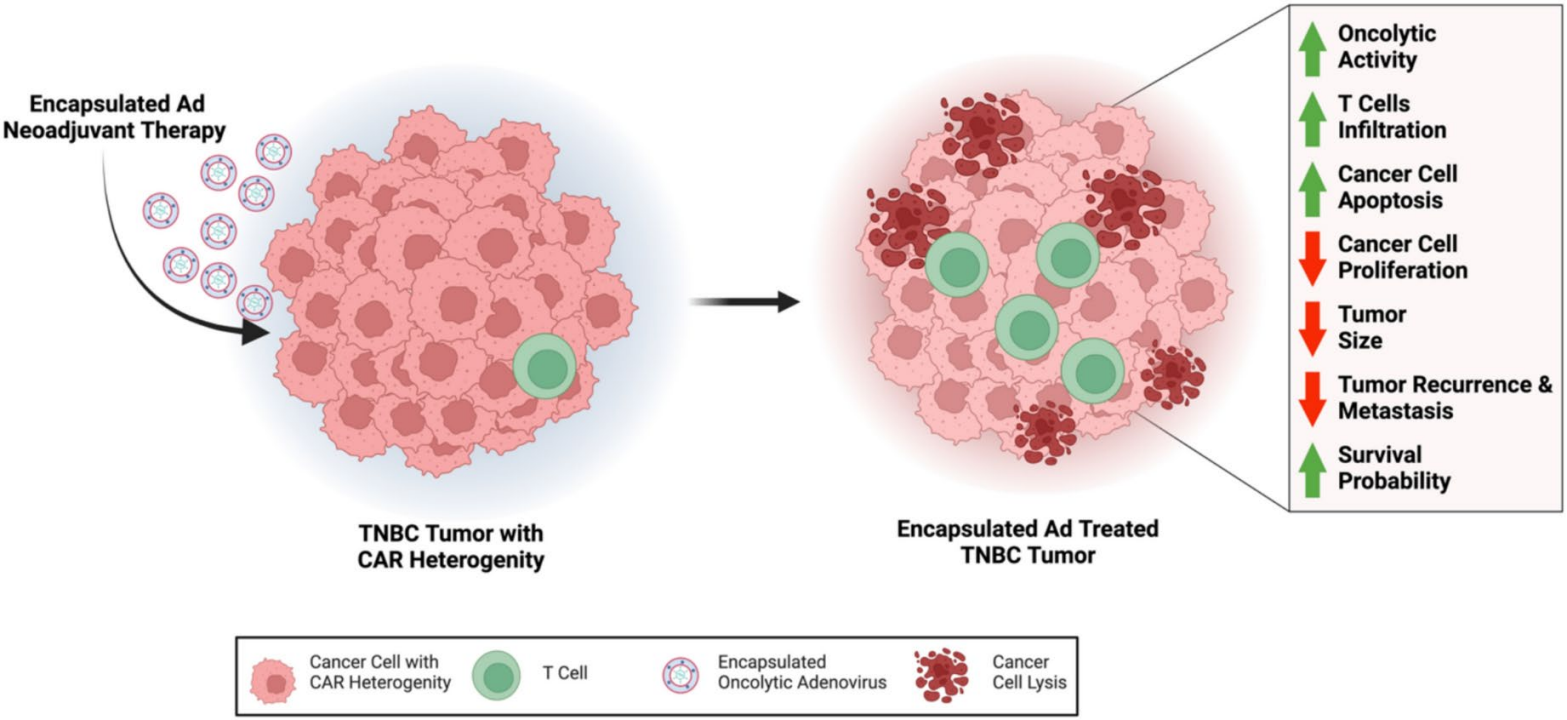
Application of encapsulated Ad-hTERT as neoadjuvant therapy for TNBC.

Ad-hTERT, also known as OBP-301, is currently undergoing evaluation in a Phase 3 clinical trial and has previously demonstrated safety and efficacy as an oncolytic adenoviral vector therapy^55–57^. In this study, we observed enhanced transduction of Ad-hTERT in TNBC cells exhibiting CAR heterogeneity facilitated by membrane fusion and folate receptor-mediated endocytosis. Furthermore, the encapsulated Ad-hTERT formulation provides multiple therapeutic advantages, highlighting its potential as a neoadjuvant therapy for TNBC tumors with CAR heterogeneity. Encapsulation significantly improves Ad-hTERT transduction efficiency, resulting in enhanced oncolytic activity. This approach also modulates the immune response by increasing T-cell infiltration and promoting a more immunologically favorable tumor microenvironment. Additionally, encapsulated Ad-hTERT induces higher levels of apoptosis and reduces tumor cell proliferation. Collectively, these effects contribute to marked tumor size reduction, decreased recurrence and metastasis, and improved survival outcomes (Fig. 9).

## Conclusions

In a neoadjuvant therapy, encapsulated Ad-hTERT completely prevented lung metastasis and primary murine tumor recurrence, which were prevalent in the control groups and the Ad-hTERT group. This led to significantly higher survival rates among mice treated with encapsulated Ad-hTERT. IHC and IF staining of primary murine tumors further supported the superior efficacy of encapsulated Ad-hTERT. In the group treated with encapsulated Ad-hTERT, higher levels of tumor apoptosis, reduced tumor proliferation, and increased T-cell infiltration were observed.

These findings highlight the potential therapeutic advantages of liposomal encapsulated Ad-hTERT over its counterpart for neoadjuvant therapy of TNBC tumors with CAR heterogeneity. Encapsulation not only enhances the apoptotic and cytotoxic effects of Ad-hTERT, but it also facilitates immune cell infiltration, reduces tumor size and recurrence, prevents metastasis, and improves survival, thereby significantly improving overall therapeutic efficacy in a CAR-low TNBC murine model.

## Materials and methods

### Reagents and cell lines

The A549, MDA-MB-436, and CCD18 Lu cell lines were purchased from the American Type Culture Collection (ATCC). The 4T1-eGFP cell line was obtained from Imanis Life Sciences. The MDA-MB-231-GFP cell line was acquired from GenTarget Inc. HCC1937, and SUM159PT cell lines were purchased from the Cell Lines Service LLC. For cell culture, Roswell Park Memorial Institute (RPMI) 1640 medium without folic acid (Gibco, Catalog #27,016,021) was supplemented with 10% Fetal Bovine Serum (FBS, Corning, Catalog #35–011-CV) and 1% Pen Strep Glutamine (PSG, Life Technologies, Catalog #10,378–016) to create complete RPMI (RP-10) for culturing 4T1-eGFP, and HCC1937 cells. Dulbecco’s Modified Eagle Medium (DMEM) with high glucose (HyClone, Catalog #SH30081.01) was supplemented with 10% FBS and 1% PSG to form complete DMEM, which was used for culturing A549 cells. A 50:50 mixture of complete RP-10 and complete DMEM was used for culturing MDA-MB-231-GFP and MDA-MB-436 cells. Ham’s F-12 (Life Technologies, Catalog #11,765,054) was supplemented with 1 g/L Bovine Serum Albumin (BSA, Sigma Aldrich Catalog #A8806), 5 mM of Ethanolamine (Sigma Aldrich, Catalog #E0135), 10 mM of HEPES (Sigma Aldrich, Catalog # H3375), 1 µg/mL of Hydrocortisone (Sigma Aldrich, Catalog #H4001), 5 µg/mL of Insulin (Sigma Aldrich, Catalog #I9278), 50 nM of Sodium Selenite (Sigma Aldrich, Catalog #S9133), 5 µg/mL of apo-Transferrin (Sigma Aldrich, Catalog #T2252), 10 nM of Triiodo Thyronine (T3, Sigma Aldrich, Catalog #T5516), and 10% FBS to prepare the complete media for the SUM159PT cell line. Eagle’s Minimum Essential Medium (EMEM) (ATCC, Catalog #30–2003) was supplemented with 10% FBS to form a complete EMEM, which was used for culturing CCD18 Lu cells. Primary human breast cell medium was prepared by using the EpiCult™-C Human Medium Kit (Stemcell™ Technologies, Catalog # 05,630), following the kit instructions for preparing the complete EpiCult™-C Medium. All cells were cultured at 37 °C and 5% CO_2_ in their respective complete media. Ad-hTERT manufacturing and purification were performed in collaboration with Vector Biolabs, Malvern, Pennsylvania, USA, as previously described^30,58^. In brief, Ad-hTERT was manufactured by cloning the hTERTp-E1A-IRES-E1B expression cassette into the Ad5/dE1/E3 vector and packaging it into adenovirus stock. The packaged viral stock was further amplified in HEK293 cells, followed by two rounds of CsCl gradient purification. The purified Ad stock was titered for both viral particles (VP) and plaque-forming units (PFU). Ad-WT was obtained from the Baylor College of Medicine.

### Human breast cell isolation and culturing

Human breast tumor cell isolation and culturing were conducted as described previously^37^. A human breast cancer specimen was obtained from a patient through the biorepository and tissue technology shared resources (BTTSR) at the University of California San Diego (UCSD). Patient consent was obtained by BTTSR, and all procedures were conducted under an Institutional Review Board-approved protocol (No. 181755). During transportation, the tumor tissue was placed in a 50 mL conical tube containing sterile phosphate buffer saline (PBS) (Fisher Scientific, Catalog # 10010072), ensuring the tissue was fully submerged. Tissue digestion was performed using the gentleMACS™ Octo Dissociator with Heaters (Miltenyi Biotec, Catalog # 130–096-427) following the protocol provided in the Human Tumor Dissociation Kit (Miltenyi Biotec, Catalog # 130-095-929). The “37C_h_TDK_3” program was selected for digestion on the Octo Dissociator instrument. Post-digestion, the tissue mixture was strained using a 100 µm strainer. The filtrate was centrifuged at 530 g for 5 minutes at room temperature to collect cells, and the supernatant was discarded. To remove red blood cells from the cell pellet, 5–10 mL of ACK buffer (Quality Biological, Catalog # 118-156-101) was added, and the mixture was incubated for 3 minutes. Cells were centrifuged again at 530 g for 5 minutes at room temperature, and the supernatant was removed. This step was repeated until no red blood cells were visible in the pellet. The resulting cell pellet contained primary human tumor cells and stromal cell subpopulations. The resultant cells were resuspended in 10 mL of PBS and centrifuged at 530 g for 5 minutes at room temperature. The final cell pellet was resuspended in 1 mL of primary human breast cell medium, and a 10 µL aliquot was used for cell counting. Cells were plated at a density of 30,000 cells per well in a 96-well plate and incubated at 37 °C with 5% CO_2_. Viral infection was performed after the cells had attached to the wells, typically 48 hours after plating.

### Manufacturing of liposome-encapsulated Ad-hTERT

Liposome encapsulation of Ad was performed as previously described^36^. Briefly, DOTAP (Avanti, Catalog # 890890C), cholesterol (Sigma-Aldrich, Catalog # C3045), PEG(2000)-PE carboxylic acid (Avanti, Catalog # 880135P), and PEG(2000)-Folate-PE (Avanti, Catalog # 880124P) were suspended in chloroform (Sigma-Aldrich, Catalog # C2432) at a molar ratio of 1:0.26:0.02:0.01. To prepare 400 µL of liposomes, the following amounts of each lipid were used: DOTAP, 387 nmol; cholesterol, 100 nmol; PEG(2000)-PE carboxylic acid, 7.01 nmol; and PEG(2000)-Folate-PE, 3 nmol, dissolved in chloroform in an amber-colored vial (Fisher Scientific, Catalog # 03-339-23C). The lipid mixture was vortexed in an amber vial for 30 minutes at ambient temperature (25 °C). The mixture was vacuum-dried overnight to form a lipid film. The dried lipid film was hydrated the following day with 400 µL of PBS (Fisher Scientific, Catalog # 10010072) while vortex-mixed. The hydrated film was stirred at 600 rpm at 4 °C for 30 minutes. Next, empty liposomes were formed by extruding the lipid solution using an Avanti Mini Extruder (Avanti, Catalog # 610000-1EA) through a 200 nm membrane (Cytiva/Whatman, Catalog # 10417004) eight times at room temperature. Ad-hTERT was added to these empty liposomes, and the suspension was incubated for 30 minutes at room temperature to yield encapsulated Ad-hTERT. The final product contained 1.4 × 10^9^ VP of Ad-hTERT/100 µL. Freshly prepared encapsulated Ad-hTERT was used for each experiment.

### In vitro transduction

A549 and CCD18 Lu cells were plated overnight at a density of 5 × 10^3^ cells/well in a 96-well plate at 37 °C and 5% CO₂ in complete media. On day 1, samples were added to the cells at a MOI of 20 (pfu per cell) and incubated at 37 °C and 5% CO₂. Analysis was performed after 3 days for A549 cells and after 5 days for CCD18 Lu cells. 4T1-eGFP, MDA-MB-231-GFP, SUM159PT, MDA-MB-436, HCC1937 cells, and patient-derived TNBC cells were plated overnight at a density of 3 × 10^4^ cells/well in a 96-well plate at 37 °C and 5% CO₂ in complete media. On day 1, samples were added to the cells at various MOIs (pfu per cell) and incubated at 37 °C and 5% CO₂. Analysis was performed after 3 days.

### GFP and DAPI fluorescence microscopy

4T1-eGFP and MDA-MB-231-GFP cells transduced with samples were analyzed under a Keyence BZ-X710 microscope (Keyence Corporation of America, IL, USA) with a GFP filter (excitation wavelength 470/40 nm, emission wavelength 525/50 nm, dichroic mirror wavelength 495 nm). After the respective transduction durations, A549, CCD18 Lu, and patient-derived primary human TNBC cells had their media removed. The cells were gently washed with PBS and fixed in cold methanol (Fisher Chemical™, Catalog # A452SK-4) for 20 minutes at -20 °C. After 20 minutes, the methanol was removed, and the cells were washed with PBS. DAPI staining (Invitrogen, Catalog # D1306) was performed according to the manufacturer’s procedure. DAPI-stained cells were analyzed under the Keyence BZ-X710 microscope with a DAPI filter (excitation wavelength 360/40 nm, emission wavelength 460/50 nm, dichroic mirror wavelength 400 nm). Cell counting was performed under the microscope (n = 3 wells), and the percentage of cell viability was calculated with reference to the PBS-treated cells.

### Cell viability assay

Following the respective transduction durations, A549, CCD18Lu, 4T1-eGFP, MDA-MB-231-GFP, and patient-derived primary TNBC cells were assessed for viability using the alamarBlue™ Cell Viability Reagent (Invitrogen, Catalog # DAL1025) in accordance with the manufacturer’s instructions. The assay was performed in triplicate (n = 3 wells), and cell viability was expressed as a percentage relative to the PBS-treated control cells.

### In vivo animal studies

All animal experiments were performed in accordance with the protocol approved by the University of California San Diego Institutional Animal Care and Use Committee (IACUC) (Protocol #S15103). All experimental procedures adhered to the Animal Research: Reporting of In Vivo Experiments (ARRIVE) guidelines. BALB/c mice were purchased from the Jackson Laboratory (Strain #000651). The mice were housed in high-efficiency particulate air cages in a specific pathogen-free facility with food and water available ad libitum and a 12-hours light/dark cycle. Female mice aged between 8 and 12 weeks were used for the experiment.

On Day 0, 1 × 10⁶ 4T1-eGFP cells in 100 µL of Matrigel^®^ (Corning, Catalog #354262) were subcutaneously injected into the right 4^th^ mammary fat pad of the mice. Tumor formation took approximately 2 weeks for physical detection. Tumor volume was determined using a caliper with the modified ellipsoidal formula: volume (mm^3^) = (width × width × length)/2.

On Day 21, when the tumor volume reached approximately 30 mm^3^, the mice were randomly divided into four groups: control PBS (n = 4), control empty liposomes (n = 4), Ad-hTERT (n = 5), and encapsulated Ad-hTERT (n = 5). Each dose was 100 µL, containing either PBS, empty liposomes (at the dose equivalent to encapsulated Ad-hTERT), 1.0 × 10⁸ PFU of Ad-hTERT in PBS, or 1.0 × 10⁸ PFU of encapsulated Ad-hTERT. For the biodistribution study, mice were sacrificed after 48 h of the treatment and tumor and liver tissues were harvested. TRIzol™ Reagent (Invitrogen, Catalog #15596026) was used for tissue digestion per the manufacturer’s protocol. Reverse transcription reactions were performed at 25 °C for 10 minutes, 37 °C for 120 minutes, 85 °C for 5 minutes, and 4°Cfor 5 minutes by cDNA Reverse Transcription Kit (Applied Biosystems, Catalog #4368813). PCR amplification was conducted with 40 cycles of denaturation at 95 °C for 20 seconds and annealing at 60 °C for 20 seconds. The threshold cycle values for E1A were obtained using a ViiA 7 real-time qPCR system (Life Technologies, Carlsbad, CA, USA) and converted to the gene copy number from the standard curves. Primers and probes were synthesized by the manufacturer (Integrated DNA Technologies, San Diego, CA, USA): E1A probe, 5'-AGCCCGAGCCAGAACCGGAGCCTGCAA-3'; E1A primers, forward, 5'-TGTGTCTGAACCTGAGCCTG-3', reverse, 5'-ATAGCAGGCGCCATTTTAGG-3'.

For a comparative therapeutic efficacy study, tumor volume was monitored every other day throughout the treatment period, with 10 intratumoral injections performed, one injection every other day to mimic the effects of a replicating virus in a human tumor model^38^. Intratumoral treatment was completed on Day 39. On Day 45, tumors were resected from the mice under anesthesia as per the approved IACUC protocol. Surgical incisions were closed with wound clips. Intraperitoneally, warm sterile saline (Cytiva Hyclone™, Catalog #Z1376) (4% of the animal’s body weight) was administered. A single injection of Ethiqa XR^®^ (buprenorphine extended-release injectable suspension, DEA Schedule CIII, NDC 86084-100-30) was administered at the recommended dose of 3.25 mg/kg subcutaneously to manage pain for up to 72 hours. All precautions were followed as per the approved Controlled Substances Use Authorization (Protocol #798), and recovery and post-operative care procedures were followed as per the approved IACUC protocol. The resected primary murine tumors were photographed, weighed, and subjected to histological analysis. After surgery, the mice recovery and survival study continued until Day 75. The recurrence of tumors at the primary site was recorded where applicable. Lungs from mice that did not survive were isolated, flash-frozen, and kept at −80 °C. On Day 75, surviving mice were sacrificed, and their lungs, along with the preserved lungs from the mice that did not survive, were isolated to study metastasis. Bright light and Dino-Lite GFP digital microscope (Dino-Lite US, Dunwell Tech, Inc., Model AM4117MTW-G2FBW) images of isolated lungs were captured and compared to the lungs isolated from healthy control mice.

### Hematoxylin and eosin (H&E) staining of primary murine tumors and lungs

Formalin-fixed paraffin-embedded (FFPE) tissue samples were heated at 60 °C for 1 hour. The samples were then cleaned and rehydrated through a series of liquid dips: three times in Clearite (Fisher, Catalog #6901TS), twice in 100% ethanol, once in 95% ethanol, and once in deionized water. H&E staining was carried out using the Thermo Gemini AS Stainer (Thermo Scientific, Waltham, MA, USA). The staining procedure involved treating the samples with Gills II Hematoxylin (Sugaripath, Catalog #1570) for 4 minutes, followed by Clarifier™ 1 (Epredia, Catalog #7401) for 5 seconds, and then with Bluing Reagent (Epredia, Catalog #7301) for 30 seconds. The samples were then washed in 70% ethanol for 1 minute and stained with Eosin (Sugaripath, Catalog #1615). This was followed by washes in 70% ethanol, 95% ethanol, and three times in 100% ethanol. Finally, the samples were washed in Clearite three times. The stained tissues were mounted using a mounting medium (VWR, Catalog #48212-154). Scanning was performed using the Aperio AT2 Digital Whole Slide Scanner (Leica, Version 102.0.4.6), and analysis was conducted with ImageScope software (Aperio, Version 12.4.0.5043).

### Caspase-3, Ki-67, CD3, and CD8 staining of primary murine tumors

FFPE cell line samples were heated at 60 °C for 1 hour. The samples were cleaned and rehydrated through a series of liquid dips: three times in xylene, twice in 100% ethanol, twice in 95% ethanol, twice in 70% ethanol, and once in deionized water. Antigen retrieval was performed in a tris-based antigen unmasking solution (citrate-based, pH 6) (Vector Laboratories, Catalog # H-3300) at 95 °C for 30 minutes. Immunofluorescent staining was carried out using an Intellipath automated IHC stainer (Biocare Medical, LLC., CA, USA). The samples were treated with Bloxall peroxidase block (Vector Laboratories, Catalog # SP-6000) for 10 minutes and washed twice with tris-buffered saline with Tween 20^®^ (TBST, Santa Cruz Biotechnology, Catalog # sc-36231-1). The tissue samples were blocked with Blocker™ BLOTTO in TBS (Thermo Scientific, Catalog # PI37530) for 10 minutes. For Ki-67 detection, the tissues were incubated with a 1:50 dilution of anti-Ki-67 antibody (GeneTex, Catalog # GTX16667). For CD3 and CD8 detection, the tissues were incubated with a 1:500 dilution of anti-CD3 antibody (Abcam, Catalog # ab16669) and anti-CD8 antibody (Invitrogen, Catalog# 14–0195-82). All three incubations were conducted for 1 hour, followed by two washes with TBST. The samples were treated with anti-rabbit HRP polymer (Cell IDX, Catalog # 2RH-50) for 30 minutes and washed twice with TBST. For Ki-67 detection, the samples were treated with Opal 570 (Akoya, Catalog # FP1488001KT) for 10 minutes. For CD3 detection, the samples were treated with Opal 690 (Akoya, Catalog # FP1497001KT) for 10 minutes. For CD8 detection, the samples were treated with Opal 480 (Akoya, Catalog # FP1500001KT) for 10 minutes. After each treatment, the samples were washed twice with TBST and then washed twice with deionized water. The tissue samples were counterstained with 1 µg/mL of DAPI (Millipore Sigma, Catalog # 10236276001) for 5 minutes. Finally, the tissues were mounted with Vectashield Vibrance coverslips (Vector Laboratories, Catalog # H-1700-10). The slides were scanned using the PhenoCycler Fusion (Akoya Biosciences, Version 2.2.0) and analyzed using Phenochart (Akoya Biosciences, Version 2.2.0).

For the detection of caspase-3, the tissue samples were incubated with a 1:200 dilution of anti-cleaved caspase-3 primary antibody (Cell Signaling, Catalog #9661S) for 1 hour. This was followed by two washes with TBST. Subsequently, the samples were treated with anti-rabbit HRP polymer (Cell IDX, Catalog # 2RH-50) for 30 minutes and washed twice with TBST. The tissue samples were then stained with DAB (brown) Chromogen (VWR, Catalog #95041-478) for 5 minutes and washed twice with deionized water. Next, they were stained with Mayer’s Hematoxylin (Sigma, Catalog # 51275-500 ml) for 5 minutes, followed by two washes with TBST and one wash with deionized water. The samples underwent dehydration and clearing and were then mounted with a xylene-based mountant. Scanning was performed using the Aperio AT2 Digital Whole Slide Scanner (Leica, Version 102.0.4.6), and analysis was conducted with ImageScope software (Aperio, Version 12.4.0.5043).

### Statistical analysis

Prism 10.4.1 software (GraphPad Software LLC, Boston, MA, USA) was used for data analysis. Comparison between the two groups was based on a two-tailed unpaired t-test. A log-rank test was performed to establish the significance of the differences between survival data. A value of p < 0.05 was determined to be statistically significant.

## Supplementary Information

Supplementary Information.

## Data Availability

The data presented in this study are available in article and supplementary material.

## Abbreviations

TNBC: Triple-negative breast cancer
Ad: Adenovirus
CAR: Coxsackievirus and adenovirus receptors
hTERT: Human telomerase reverse transcriptase
Ad-hTERT: Adenovirus with human telomerase reverse transcriptase
USFDA: United States food and drug administration
GFP: Green fluorescent protein
RPMI: Roswell park memorial institute
FBS: Fetal bovine serum
PSG: Pen strep glutamine
DMEM: Dulbecco’s modified eagle medium
EMEM: Eagle’s minimum essential medium
VP: Viral particles
PFU: Plaque-forming units
Ad-WT: Wild type adenovirus
BTTSR: Biorepository and tissue technology shared resources
UCSD: University of California San Diego
PBS: Phosphate buffer saline
MOI: Multiplicity of infection
IACUC: Institutional animal care and use committee
H&E: Hematoxylin and eosin
FFPE: Formalin-fixed paraffin-embedded
IRES: Internal ribosome entry site
