# Supplementary material for: Liposomal oncolytic adenovirus as a neoadjuvant therapy for triple-negative breast cancer
Source: Sci Rep. 2025 May 14;15:16737. doi: 10.1038/s41598-025-00211-2 (PMC12078467; doi:10.1038/s41598-025-00211-2)
Supplement: Supplementary file 1 — Supplementary Information. [file 41598_2025_211_MOESM1_ESM.pdf]

**Supplementary Information**

# Liposomal Oncolytic Adenovirus as a Neoadjuvant Therapy for Triple Negative Breast Cancer

*Jaimin R. Shah[1, 2, 3], Tao Dong[1, 2, 4], Abraham T. Phung[1, 2, 4], Sohini Khan[1, 5], Omonigho Aisagbonhi[1, 6], Sarah L. Blair[1, 5], Michael Bouvet[1, 5], William C. Trogler[2], and Andrew C. Kummel[1, 2*]*

[1]Moores Cancer Center, University of California San Diego, La Jolla, California 92093, USA

[2]Department of Chemistry & Biochemistry, University of California San Diego, La Jolla, California 92093, USA

[3]Program in Materials Science and Engineering, University of California San Diego, La Jolla, California 92093, USA

[4]Department of NanoEngineering, University of California San Diego, La Jolla, California 92093, USA

[5]Department of Surgery, University of California San Diego, La Jolla, California 92093, USA

[6] Department of Pathology, University of California San Diego, La Jolla, California 92093, USA

* Corresponding Author Email: akummel@ucsd.edu

# Supplementary Information

## Specificity of Ad-hTERT

To confirm the specificity of Ad-hTERT, it was compared with Ad-WT in both TERT-positive and TERT-negative cell lines. TERT-positive A549 cells, an epithelial cell line derived from lung adenocarcinoma, and TERT-negative CCD 18Lu cells, fibroblast cells isolated from the lung, were used for this purpose. A549 cells and CCD 18Lu were infected at a multiplicity of infection (MOI) of 20 for a period of 3 and 5 days, respectively (n = 3). DAPI-stained fluorescence microscopy images showed that cytotoxicity of Ad-hTERT was specific to the TERT-positive A549 cells (**Figure S1A**). Compared to PBS-treated control cells, Ad-hTERT was found to be cytotoxic to A549 cells, whereas cytotoxicity was not observed in TERT-negative CCD 18Lu cells (**Figure S1A**). In contrast, Ad-WT was found to be cytotoxic to both TERT-positive and TERT-negative cells. The comparative quantitative percentage cell viability of Ad-hTERT and Ad-WT in TERT-positive A549 cells and TERT-negative CCD 18Lu cells using alamarBlue™, highlights the specificity of Ad-hTERT in contrast to Ad-WT (**Figure S1B**).

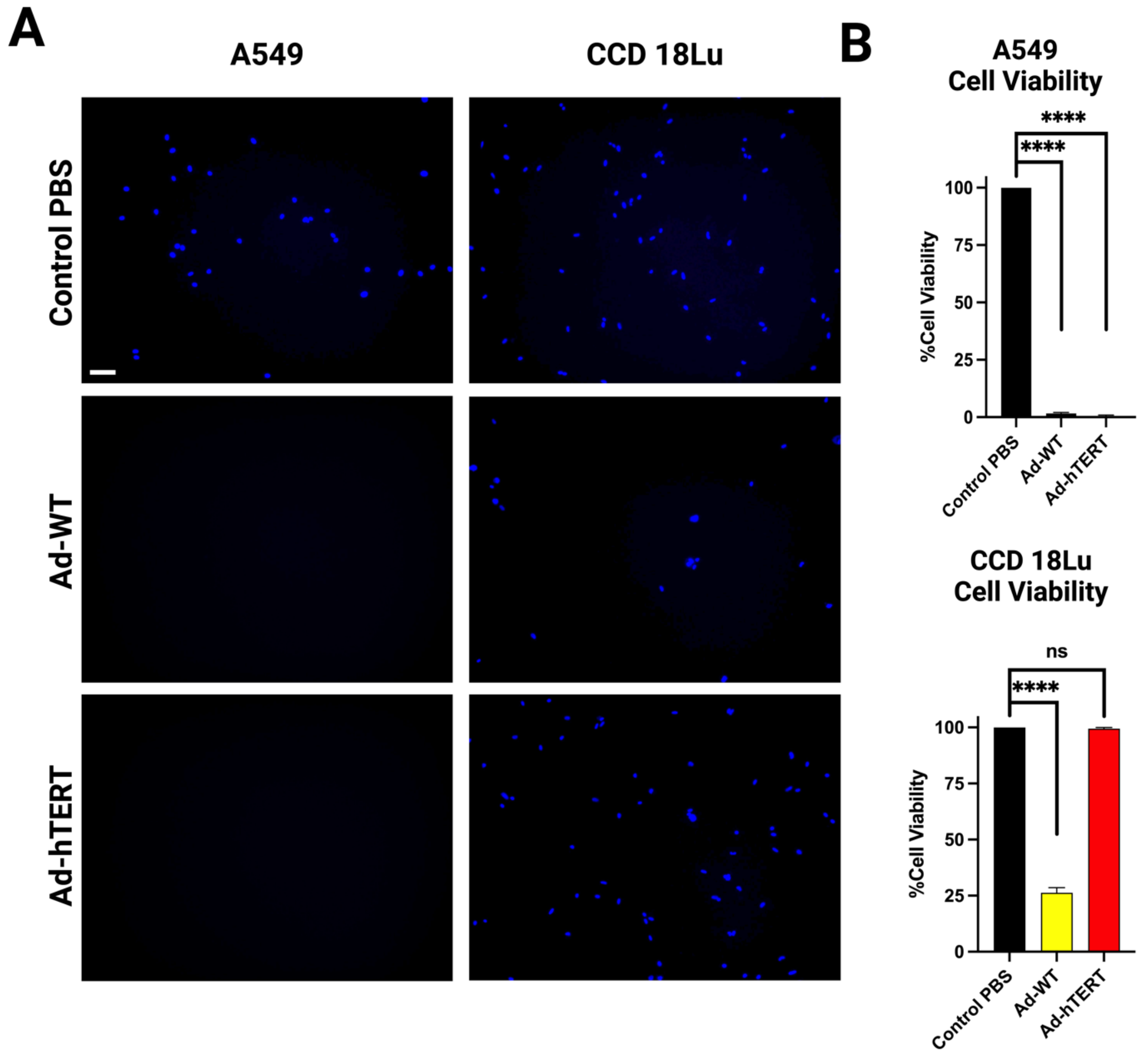


**Figure S1.** Comparison of cytopathic effects of Ad-hTERT, and Ad-WT in TERT-positive (A549) and TERT-negative (CCD 18Lu) cells. (A) DAPI-stained fluorescence microscopy images comparing Ad-hTERT and Ad-WT effects on TERT-positive A549 cells and TERT-negative CCD 18Lu cells at a multiplicity of infection (MOI) of 20 and a transduction duration of 3 and 5 days respectively. Images were captured at 100× magnification. Scale bar = 100 µm. (B) Comparative percentage cell viability exposed to Ad-hTERT (red bar) and Ad-WT (yellow bar) in TERT-positive A549 cells and TERT-negative CCD 18Lu cells at an MOI of 20 and a transduction duration of 5 days (n = 3) using alamarBlue™. Ad-WT was non-specific to cancer cells, lysing both TERT-positive A549 and TERT-negative CCD 18Lu cells. In contrast, Ad-hTERT was specific to cancer cells, consistent with preferentially lysing TERT-positive A549 cells, while sparing TERT-negative CCD 18Lu cells.

# Supplementary Information

## CAR Expression Analysis by Immunofluorescence

Formalin-fixed paraffin-embedded (FFPE) cell line samples were heated at 60 °C for 1 hour. The samples were cleaned and rehydrated through a series of liquid dips: three times in xylene, twice in 100% ethanol, twice in 95% ethanol, twice in 70% ethanol, and once in deionized water. Antigen retrieval was performed in a tris-based antigen unmasking solution (citrate-based, pH 6) (Vector Laboratories, Catalog # H-3301) at 95 °C for 30 minutes. Immunofluorescent staining was carried out using an Intellipath automated IHC stainer (Biocare Medical, LLC., CA, USA). The samples were treated with Bloxall peroxidase block (Vector Laboratories, Catalog # SP-6000) for 10 minutes and washed twice with tris-buffered saline with Tween 20® (TBST, Santa Cruz Biotechnology, Catalog # sc-36231-1). The tissue samples were blocked with Blocker™ BLOTTO in TBS (Thermo Scientific, Catalog # PI37530) for 10 minutes. The tissues were incubated with a 1:50 dilution of Anti-CAR Polyclonal Antibody (Invitrogen, Catalog # PA5-110995) for 1 hour, followed by two washes with TBST. The samples were treated with anti-rabbit HRP polymer (Cell IDX, Catalog # 2RH-50) for 30 minutes and washed twice with TBST. The samples were treated with Opal 620 (Akoya, Catalog # FP1495001KT) for 10 minutes and washed twice with TBST, followed by two washes with deionized water. The tissue samples were counterstained with 1 μg/mL of DAPI (Millipore Sigma, Catalog # 10236276001) for 5 minutes. Finally, the tissues were mounted with Vectashield Vibrance cover slips (Vector Laboratories, Catalog # H-1700-10). The slides were scanned using the PhenoCycler Fusion (Akoya Biosciences, Version 2.2.0).

# Supplementary Information

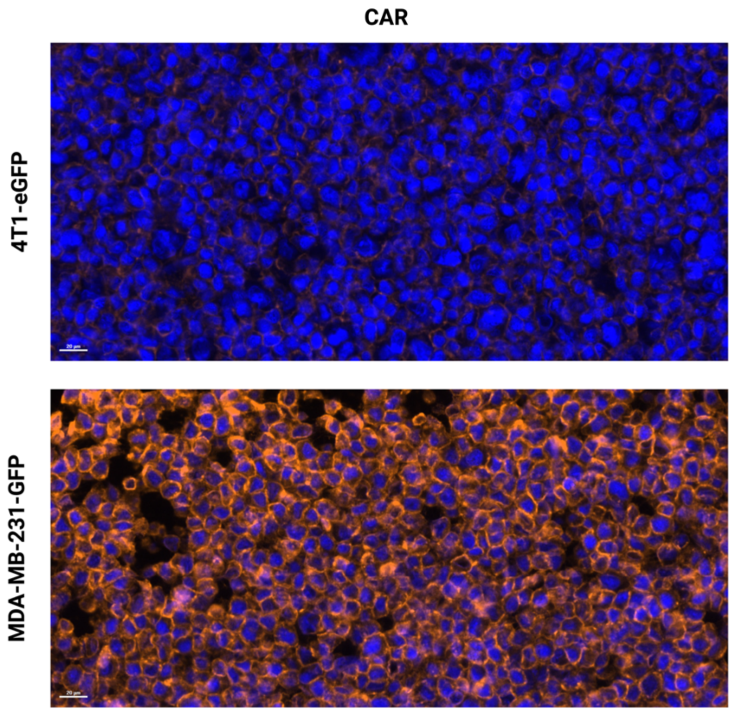


**Figure S2**. Immunofluorescence Histology for CAR. Blue = DAPI, Orange = CAR. Scale bar = 20 μm. 4T1-eGFP cells are CAR-low and MDA-MB-231-GFP cells are CAR-high.

# Supplementary Information

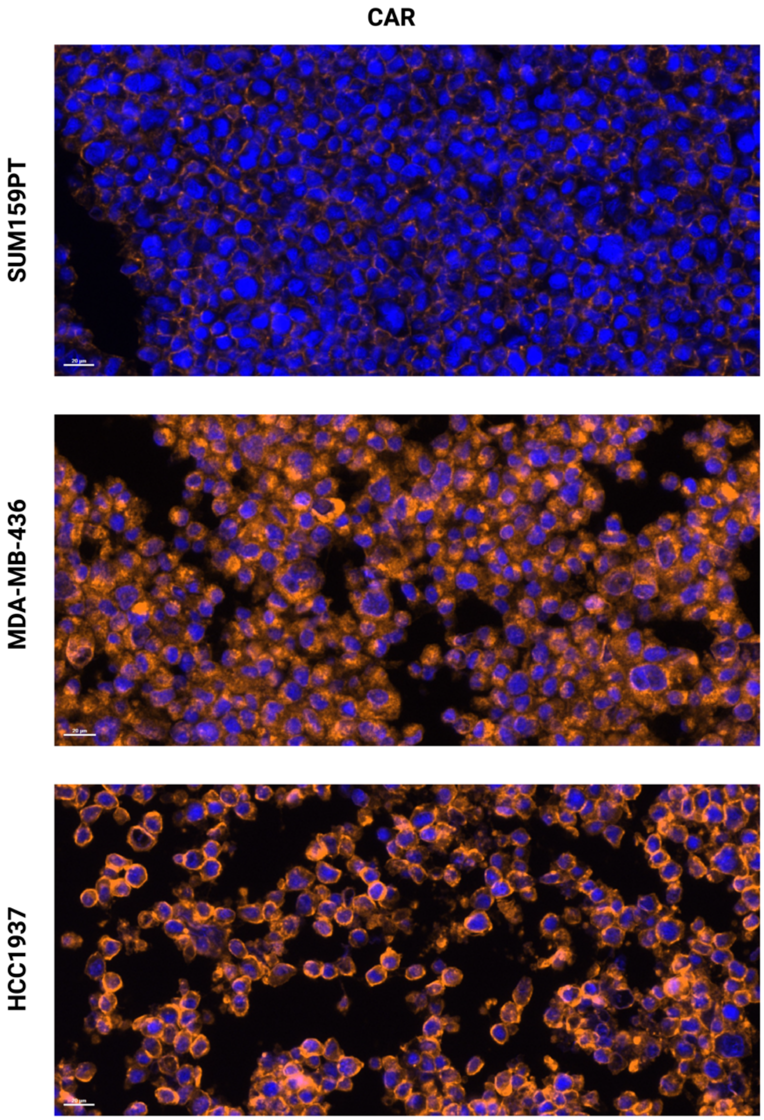


**Figure S3** Immunofluorescence Histology for CAR. Blue = DAPI, Orange = CAR. Scale bar = 20 μm. SUM159PT cells were identified as CAR-low. MDA-MB-436, and HCC1937 cells were identified as CAR-high.

Supplementary Information

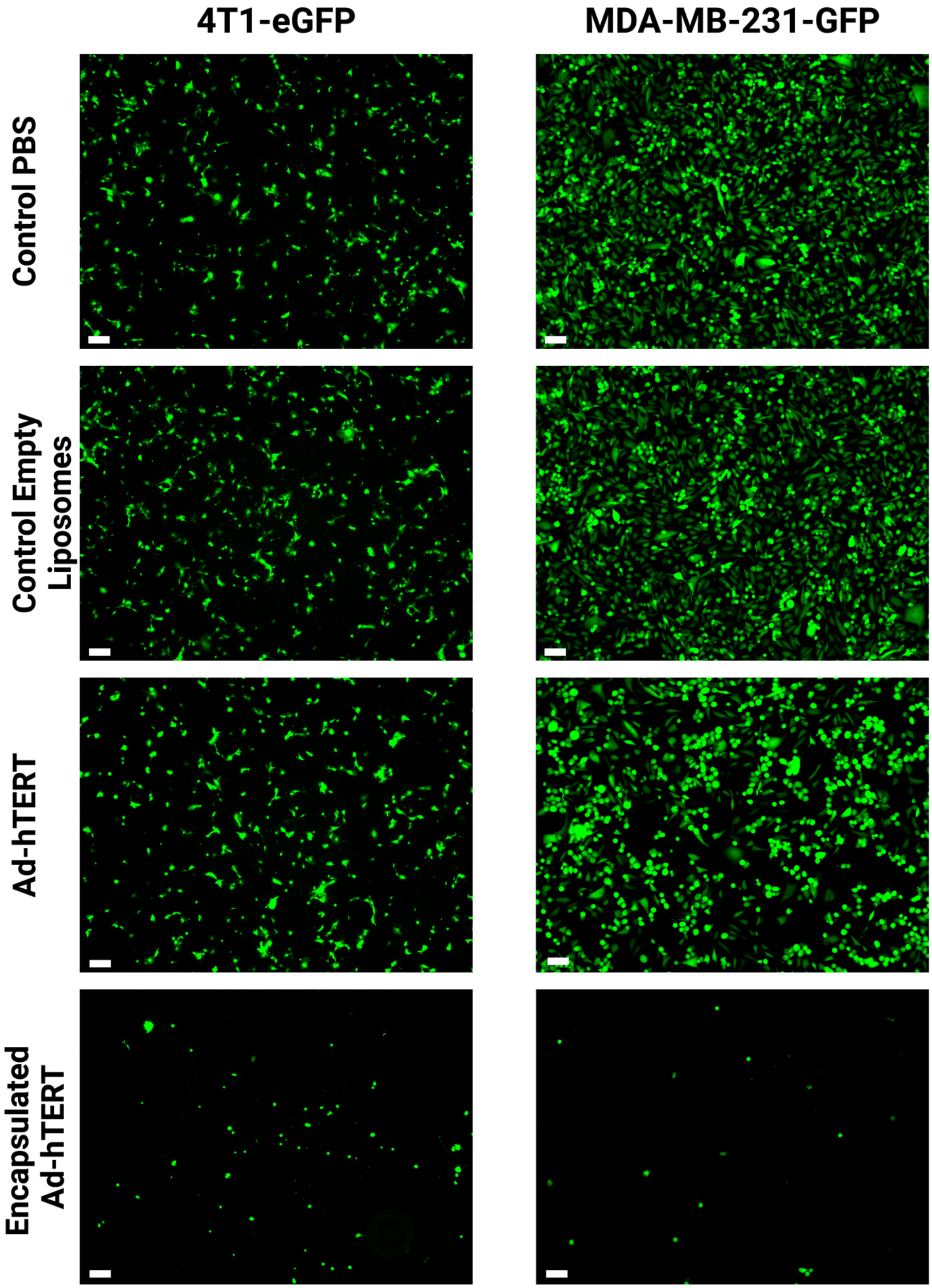

**Figure S4:** Comparative GFP fluorescence microscopy images of Ad-hTERT and encapsulated Ad-hTERT on 4T1-eGFP TNBC and MDA-MB-231-GFP TNBC cells at an MOI of 100 and a transduction time of 72 hours. Images were captured at 40× magnification. Scale bar = 200 µm

## Dynamic Light Scattering (DLS) and Zeta Potential Measurements

Mean particle sizes of sample formulations were determined using a Malvern Zetasizer Nano-ZS (Malvern Pananalytical, Malvern, UK). Prior to measurement, samples were diluted with PBS (1:10). For DLS, five acquisitions were taken at 10 s each. The system was used in the auto measuring mode. Mean hydrodynamic size, and Polydispersity Index (PDI) were automatically calculated using the Malvern's Zetasizer software 8.02 (Malvern Pananalytical, Malvern, UK). For zeta potential, the system was used in the auto measuring mode. Minimum five and maximum fifteen acquisitions were taken with forty-five seconds delay between measurements. Mean zeta potential was automatically calculated using the Malvern's Zetasizer software. The samples were analyzed in triplicates (n = 3).

**Table S1**. Particle size (z-average) using DLS, and zeta potential of Ad-hTERT and encapsulated Ad-hTERT (n = 3).

| Formulation | z-Average (nm) | Polydispersity Index (PDI) | Zeta Potential (mV) |
|---|---|---|---|
| Ad-hTERT | 115 ± 0 | 0.08 ± 0.00 | –2.75 ± 0.21 |
| Empty liposomes | 186 ± 5 | 0.19 ± 0.02 | 1.54 ± 0.61 |

## Supplementary Information

| Formulation | z-Average (nm) | Polydispersity Index (PDI) | Zeta Potential (mV) |
|---|---|---|---|
| Encapsulated Ad-hTERT | 195 ± 4 | 0.18 ± 0.05 | −1.87 ± 0.43 |

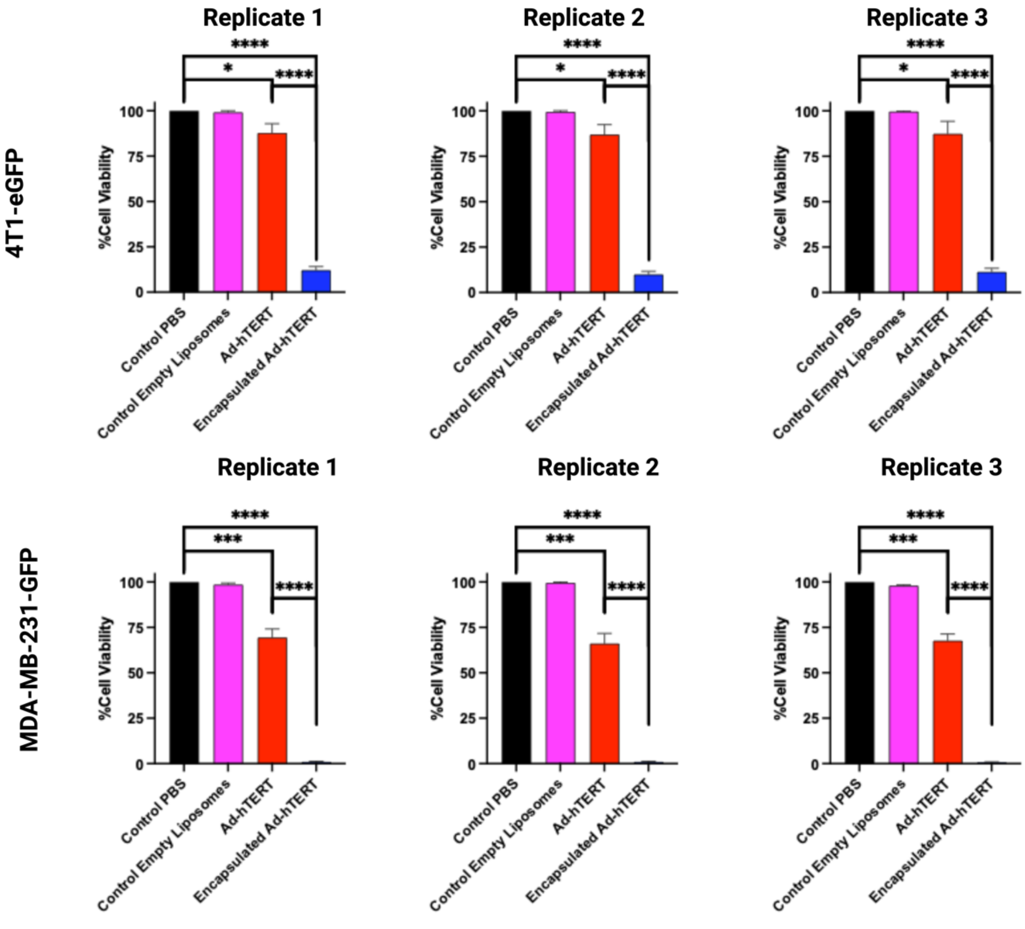


**Figure S5.** Experimental replicates - comparative percentage cell viability using alamarBlue™.

Ad-hTERT (red bar) and encapsulated Ad-hTERT (blue bar) on 4T1-eGFP TNBC, MDA-MB-231-GFP TNBC cells, at an MOI of 100 and a transduction time of 72 hours (n = 3).

# Supplementary Information

Experimental replicates were independently performed from start to finish to demonstrate the reproducibility of the assay. For all replicates, encapsulated Ad-hTERT demonstrated significantly higher cytotoxicity (p-value: **** ≤ 0.0001) compared to the encapsulated Ad-hTERT.

## Confirmation of Folate Receptor-Mediated Transduction

For cell culture, Roswell Park Memorial Institute (RPMI) 1640 medium without folic acid (Gibco, Catalog #27016021) was supplemented with 10% Fetal Bovine Serum (FBS, Corning, Catalog #35-011-CV) and 1% Penicillin-Streptomycin-Glutamine (PSG, Life Technologies, Catalog #10378-016) to prepare complete RPMI (RP-10) for culturing 4T1-eGFP cells. These cells were designated as folic acid-negative media-treated cells. For the folic acid-positive condition, folic acid was added to the complete media at a final concentration of 1 mg/L (Thermo Scientific Chemicals, Catalog #216630100).

4T1-eGFP cells were plated overnight at a density of $3 \times 10^4$ cells/well in a 96-well plate and incubated at 37 °C with 5% $CO_2$ in complete media. On Day 1, samples were added to the cells at a multiplicity of infection (MOI) of 100 (pfu per cell) and incubated under the same conditions. Analysis was performed after 3 days.

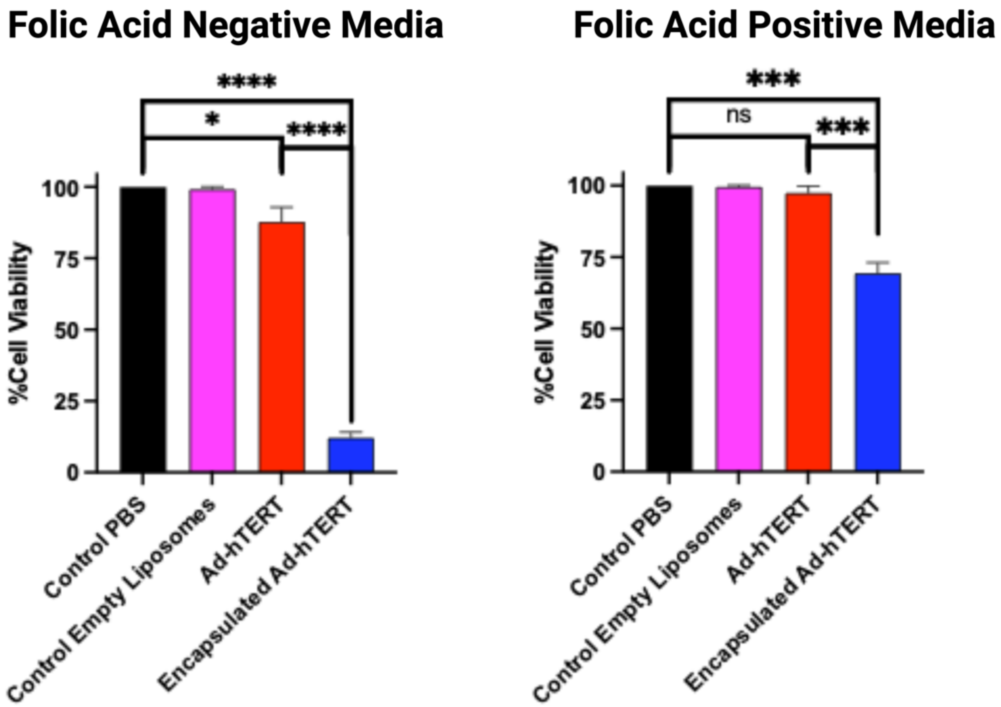


**Figure S6.** Comparative percentage cell viability of 4T1-eGFP cells cultured in RPMI 1640 media with and without folic acid, measured using alamarBlue™.
The results demonstrate significantly lower cytotoxicity with encapsulated Ad-hTERT in media containing folic acid, further supporting that the increased transduction efficiency observed with DOTAP-folate liposomes is due to folate receptor-mediated endocytosis.

**Hematoxylin and Eosin (H&E) Staining of Primary Tumors**

FFPE tissue samples were heated at 60 °C for 1 hour. The samples were then cleaned and rehydrated through a series of liquid dips: three times in Clearite (Fisher, Catalog #6901TS), twice in 100% ethanol, once in 95% ethanol, and once in deionized water. Hematoxylin and eosin (H&E) staining was carried out using the Thermo Gemini AS Stainer (Thermo Scientific, Waltham, MA, USA). The staining procedure involved treating the samples with Gills II Hematoxylin (Sugaripath, Catalog #1570) for 4 minutes, followed by Clarifier™ 1 (Epredia, Catalog #7401) for 5 seconds, and then with Bluing Reagent (Epredia, Catalog #7301) for 30

seconds. The samples were then washed in 70% ethanol for 1 minute and stained with Eosin (Sugaripath, Catalog #1615). This was followed by washes in 70% ethanol, 95% ethanol, and three times in 100% ethanol. Finally, the samples were washed in Clearite three times. The stained tissues were mounted using mounting medium (VWR, Catalog #48212-154). Scanning was performed using the Aperio AT2 Digital Whole Slide Scanner (Leica, Version 102.0.4.6), and analysis was conducted with ImageScope software (Aperio, Version 12.4.0.5043).

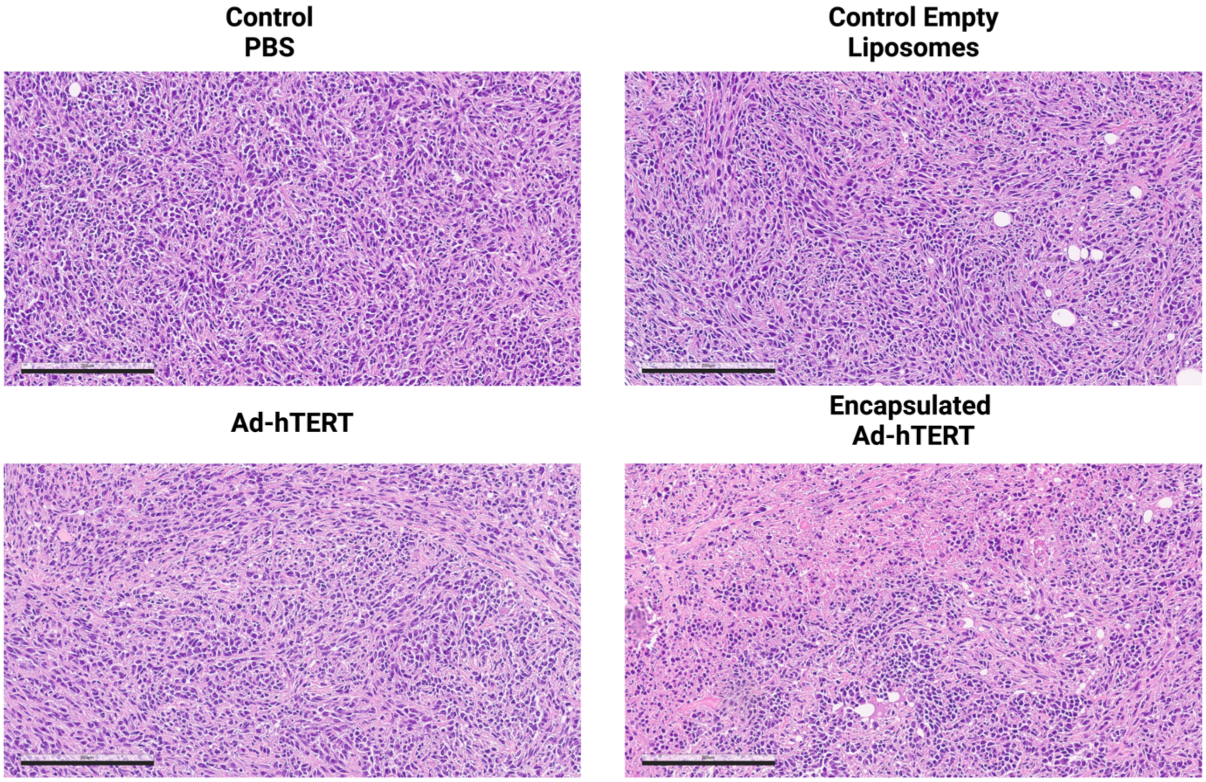


**Figure S7**. H&E staining of primary mouse tumors. Blue = nuclei, Bright pink to red = erythrocytes and eosinophilic granules, Various shades of pink = cytoplasm. Scale bar = 200 μm
